# Cross-dehydrogenative coupling for the intermolecular C–O bond formation

**DOI:** 10.3762/bjoc.11.13

**Published:** 2015-01-20

**Authors:** Igor B Krylov, Vera A Vil’, Alexander O Terent’ev

**Affiliations:** 1 N. D. Zelinsky Institute of Organic Chemistry, Russian Academy of Sciences, Leninsky Prospect 47, Moscow, 119991, Russia

**Keywords:** acyloxylation, alkoxylation, C–H functionalization, C–O bond formation, cross-dehydrogenative coupling, oxidative cross-coupling

## Abstract

The present review summarizes primary publications on the cross-dehydrogenative C–O coupling, with special emphasis on the studies published after 2000. The starting compound, which donates a carbon atom for the formation of a new C–O bond, is called the CH-reagent or the C-reagent, and the compound, an oxygen atom of which is involved in the new bond, is called the OH-reagent or the O-reagent. Alcohols and carboxylic acids are most commonly used as O-reagents; hydroxylamine derivatives, hydroperoxides, and sulfonic acids are employed less often. The cross-dehydrogenative C–O coupling reactions are carried out using different C-reagents, such as compounds containing directing functional groups (amide, heteroaromatic, oxime, and so on) and compounds with activated C–H bonds (aldehydes, alcohols, ketones, ethers, amines, amides, compounds containing the benzyl, allyl, or propargyl moiety). An analysis of the published data showed that the principles at the basis of a particular cross-dehydrogenative C–O coupling reaction are dictated mainly by the nature of the C-reagent. Hence, in the present review the data are classified according to the structures of C-reagents, and, in the second place, according to the type of oxidative systems. Besides the typical cross-dehydrogenative coupling reactions of CH- and OH-reagents, closely related C–H activation processes involving intermolecular C–O bond formation are discussed: acyloxylation reactions with ArI(O_2_CR)_2_ reagents and generation of O-reagents in situ from C-reagents (methylarenes, aldehydes, etc.).

## Introduction

**Scheme 1:**
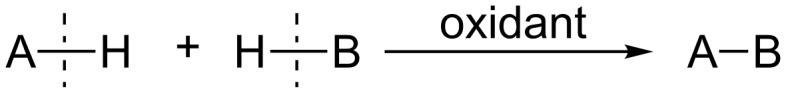
Cross-dehydrogenative coupling.

The development of methods for the cross-dehydrogenative coupling (CDC; or oxidative cross coupling) is an important field of modern organic chemistry. These terms commonly refer to reactions, in which two different molecules are linked by a new bond accompanied by the elimination of a hydrogen atom from each molecule (Scheme 1) [1–15]; however, these terms are also applied to a large number of various reactions with oxidants, which involve the intermolecular formation of new bonds between the starting molecules. For instance, such reactions involve the oxidation of several C–H bonds, the elimination not only of hydrogen atoms but also of other moieties from the starting molecules, the addition at C–C multiple bonds, and so on.

The cross-dehydrogenative coupling can be employed to form a new bond with high atomic efficiency and does not require additional synthetic steps for the introduction of functional groups (for example, such as -Hal, -OTf, -BR_2_, -SnR_3_, -SiR_3_, -ZnHal, -MgHal) into molecules necessary in other cross-coupling reactions. Therefore, the cross-dehydrogenative coupling is a promising approach to the minimization of byproduct formation and the reduction of the number of steps of the organic synthesis [1–15].

Studies of the cross-dehydrogenative coupling are not only of practical but also of fundamental interest because new aspects of the reactivity of organic compounds have to be found for the performance of these reactions. The prediction of the conditions necessary for the efficient cross-dehydrogenative coupling is an important problem that requires an understanding of the mechanisms of these processes.

**Scheme 2:**
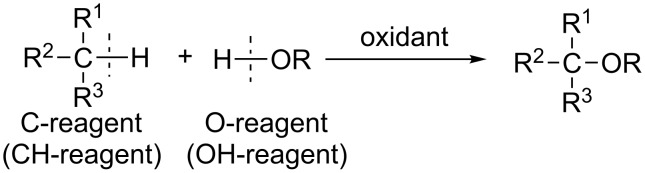
Cross-dehydrogenative C–O coupling.

Among cross-dehydrogenative coupling reactions, C–C coupling reactions have been studied in most detail [1–14], whereas the C–O coupling is less well-known (Scheme 2). We present the first systematic review of the main approaches to the cross-dehydrogenative C–O coupling. The starting compound, which donates a carbon atom for the formation of a new C–O bond, is called the CH-reagent or the C-reagent, and the compound, an oxygen atom of which is involved in the new bond, is called the OH-reagent or the O-reagent.

Alcohols and carboxylic acids are most commonly used as O-reagents; hydroxylamine derivatives, hydroperoxides, and sulfonic acids are employed less frequently. In the case of O-reagent PhI(O_2_CR)_2_, dehydrogenated carboxylic acid RCO_2_H is preliminary included in the oxidant, so C–H acyloxylation with PhI(O_2_CR)_2_ can be considered as the second stage in a two-step cross-dehydrogenative C–O coupling. The formation of a new C–O bond generally takes place with the involvement of an O-nucleophile, an O-radical, or an O-electrophile. In the oxidative coupling with O-reagents as nucleophiles, electrophilic intermediates that are generated from C-reagents are prone to side transformations. Therefore, O-reagents are often used in excess amounts. The C–O coupling reactions involving O-centered radicals are generally performed under severe conditions. In addition, O-radicals are highly reactive and unstable, and the reactions with these radicals are often non-selective and are accompanied by the formation of alcohols, carbonyl compounds, and fragmentation products. The examples of the C–O bond formation between two molecules using O-electrophiles are rare; electron-deficient peroxides with a specific structure can act as O-electrophiles [16–18]. These processes are not consistent with general Scheme 2 of the cross-dehydrogenative C–O coupling and are not considered in the present review.

The cross-dehydrogenative C–O coupling reactions are carried out using different C-reagents, such as compounds containing directing functional groups (amide, heteroaromatic, oxime, and so on) and compounds with activated C–H bonds (aldehydes, alcohols, ketones, ethers, amines, amides, compounds containing a benzyl, allyl, or propargyl moiety). An analysis of the published data showed that the principles at the basis of a particular cross-dehydrogenative C–O coupling reaction are dictated mainly by the nature of the C-reagent. Hence, in the present review the data are classified according to the structures of C-reagents, and, in the second place, according to the type of oxidative systems. Since structurally different OH-reagents are often involved in C–O coupling reactions of the same type, the classification according to the structures of O-reagents is inconvenient and was not applied.

Some cross-dehydrogenative C–O coupling reactions are cursorily described in reviews on the oxidative C–heteroatom bond formation without the use of metal compounds [15], the Pd(II)-catalyzed oxidative C–C, C–O, and C–N bond formation [3], the transition metal-catalyzed etherification of unactivated C–H bonds [19], the Pd(II)-catalyzed oxidative functionalization at the allylic position of alkenes [20–21], the oxidative functionalization catalyzed by copper compounds to form C–C, C–N, C–O, C–Hal, C–P, and N–N bonds [10], the Bu_4_NI/*t-*BuOOH oxidative system [22], selective functionalization of molecules [23], the oxidative esterification and oxidative amidation of aldehydes [24], and the transition metal-catalyzed radical oxidative cross-couplings [13].

The present review summarizes primary publications on the cross-dehydrogenative C–O coupling, with special emphasis on the studies published after 2000. The focus is on the reactions described by general Scheme 2.

## Review

### 1 C-Reagents containing directing groups in cross-dehydrogenative C–O coupling

Nitrogen-containing moieties (amide, pyridine, oxime, etc.) are most commonly used as directing groups, which are responsible for the regioselectivity of the C–O coupling. Most transformations of this type are catalyzed by Pd(II) compounds. Examples of the use of copper and ruthenium compounds as catalysts were also reported. It is commonly assumed that the reaction proceeds via the C–metal bond formation accompanied by the C–H bond cleavage assisted by the directing group of the substrate, which forms a complex with the metal ion. The mechanism of this type of reactions was considered in detail in the publications [25–32].

#### 1.1 Reactions involving C(sp^2^)–H bonds of aromatic C-reagents

In one of the first publications on the preparative introduction of the –OR group into CH-reagents containing directing groups, 8-methylquinoline, 2-arylpyridines, *N*-phenylpyrazole, azobenzene, and benzylideneaniline were subjected to the acetoxylation using the Pd(OAc)_2_/PhI(OAc)_2_ system [33]. More recently, reactions involving the same and some other directing groups were studied in more detail. In most of the studies, Pd(OAc)_2_ was used as the catalyst, and PhI(OAc)_2_ or peroxides served as the oxidants.

**Scheme 3:**
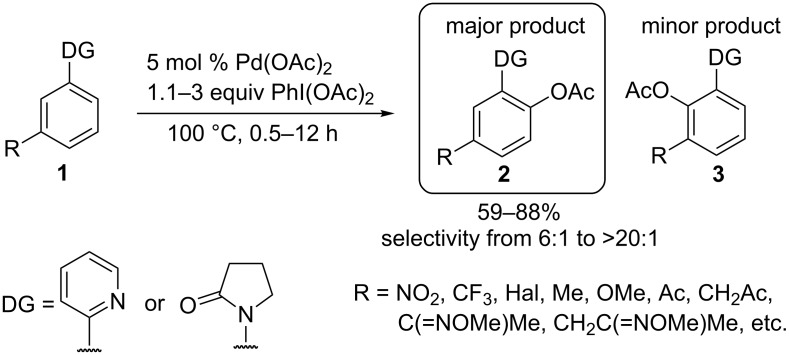
Regioselective *ortho*-acetoxylation of *meta*-substituted arylpyridines and *N*-arylamides.

The regioselectivity of the *ortho*-acetoxylation of *meta*-substituted arylpyridines and *N*-arylamides **1** was studied [34]. The acetoxylation occurs mainly at the sterically more accessible *para*-position relative to the substituent R to form product **2**. The lowest regioselectivity (**2**:**3** = 6:1) was observed in the case of R = F (Scheme 3).

The pyridine moiety served as the directing group in many works to accomplish the *ortho*-acyloxylation of arenes **4** giving products **5** (Table 1). The reactions were catalyzed by copper, palladium, or rhodium salts. Carboxylic acids or their salts, as well as aldehydes, methylarenes, arylethylenes, and arylacetylenes were used as precursors of the acyloxy fragment.

**Table 1:** *ortho*-Acyloxylation of arenes **4** directed by pyridine moiety.

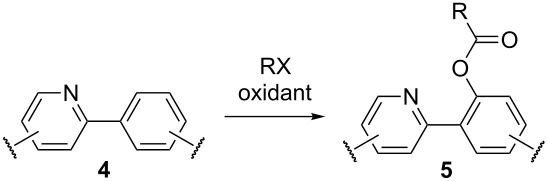			

Conditions	RX	Yield of **5**, %	Ref.

[Rh(cod)Cl]_2_ (5 mol %) P(Cyclohexyl)_3_·HBF_4_ (7.5 mol %) CuI (40 mol %) phenanthroline (10 mol %) *N*-methylpyrrolidone, 130 °C, 36 h	RCOOH (0.5 or 2 equiv); R = Ar, CH=CHPh, Me	43–85	[35]
Pd(OAc)_2_ (10 mol %) CuI (1 equiv) Ag_2_CO_3_ (1 equiv) O_2_ dichloroethane, 80 °C	RCOOH; R = Ar, Me	53–78	[36]
Cu(OTf)_2_ K_2_S_2_O_8_ toluene, 130 °C, 24 h	RCOONa; R = Ar	35–86	[37]
Cu(OAc)_2_ (10 mol %) *t-*BuOOH (2–4 equiv) PhCl or without a solvent, 135 °C, 24–40 h	RCHO or ArCH_3_; R = Ar, *n*-Bu, *n*-Pr	20–56	[38]
Cu(OAc)_2_ (20 mol %) *t-*BuOOH (10 moles per mole of arene) PhCl, 120 °C, 10–22 h	ArCH=CH_2_ or ArC≡CH (2 equiv)	48–81	[39]

The cross-dehydrogenative C–O coupling with 2-arylpyridines **4** proceeds also in the presence of the Cu(OAc)_2_/O_2_ system [40] and under electrochemical oxidation in the presence of Pd(II) salts [41].

**Scheme 4:**
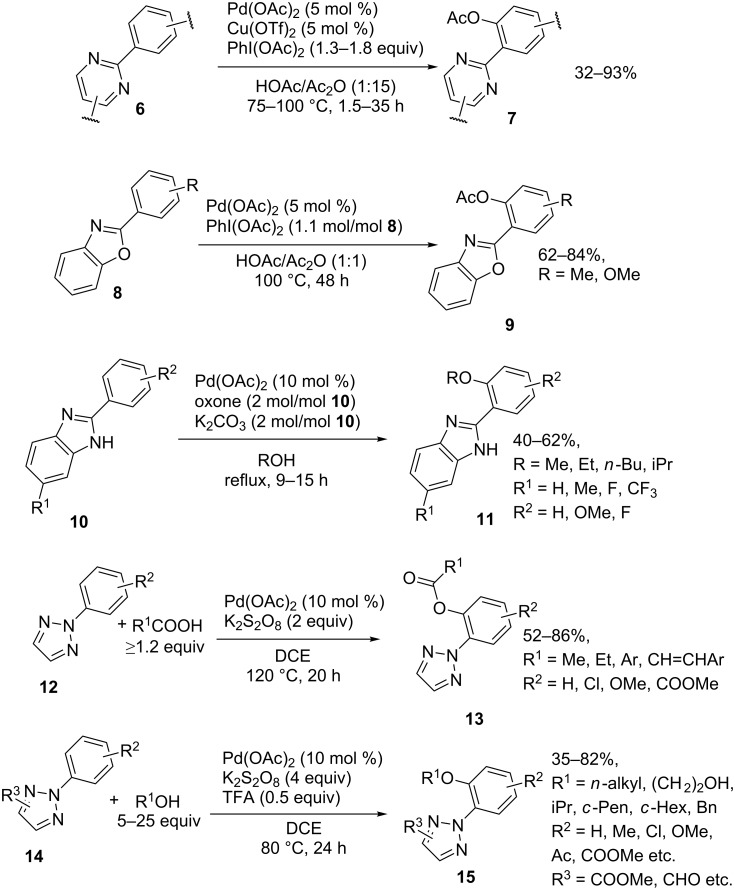
*ortho-*Acyloxylation and alkoxylation of arenes directed by pyrimidine, benzoxazole, benzimidazole and triazole groups.

The pyrimidine (acetoxylation of **6** to form **7**) [42], benzoxazole (acetoxylation of **8** to form **9**) [43], benzimidazole (alkoxylation of **10** to form **11**) [44], and triazole (acyloxylation of **12** to form **13** [45], alkoxylation of **14** to form **15** [46]) moieties were also used as directing groups for the *ortho-*acyloxylation and alkoxylation of arenes (Scheme 4).

**Scheme 5:**
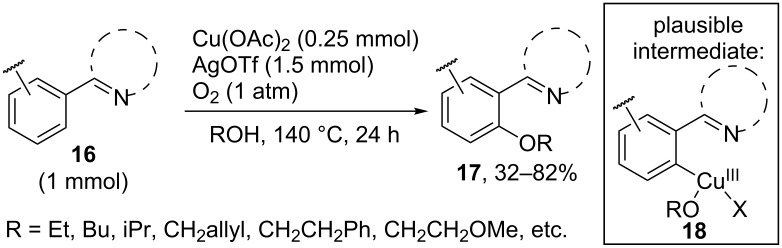
Cu(OAc)_2_/AgOTf/O_2_ oxidative system in the *ortho*-alkoxylation of arenes.

The pyridine, pyrimidine, or pyrazole moiety serves as the directing group in the oxidative *ortho*-alkoxylation of arenes **16** with the Cu(OAc)_2_/AgOTf/O_2_ system giving coupling products **17** (Scheme 5) [47]. It is supposed that copper is inserted into the C–H bond of arene, the resulting Cu(II) complex is oxidized by silver(I) ions to Cu(III) complex **18**, and the C–O bond is formed via reductive elimination. The drawbacks of this method are the use of large amounts of silver triflate and alcohol and the high temperature of the reaction.

**Scheme 6:**
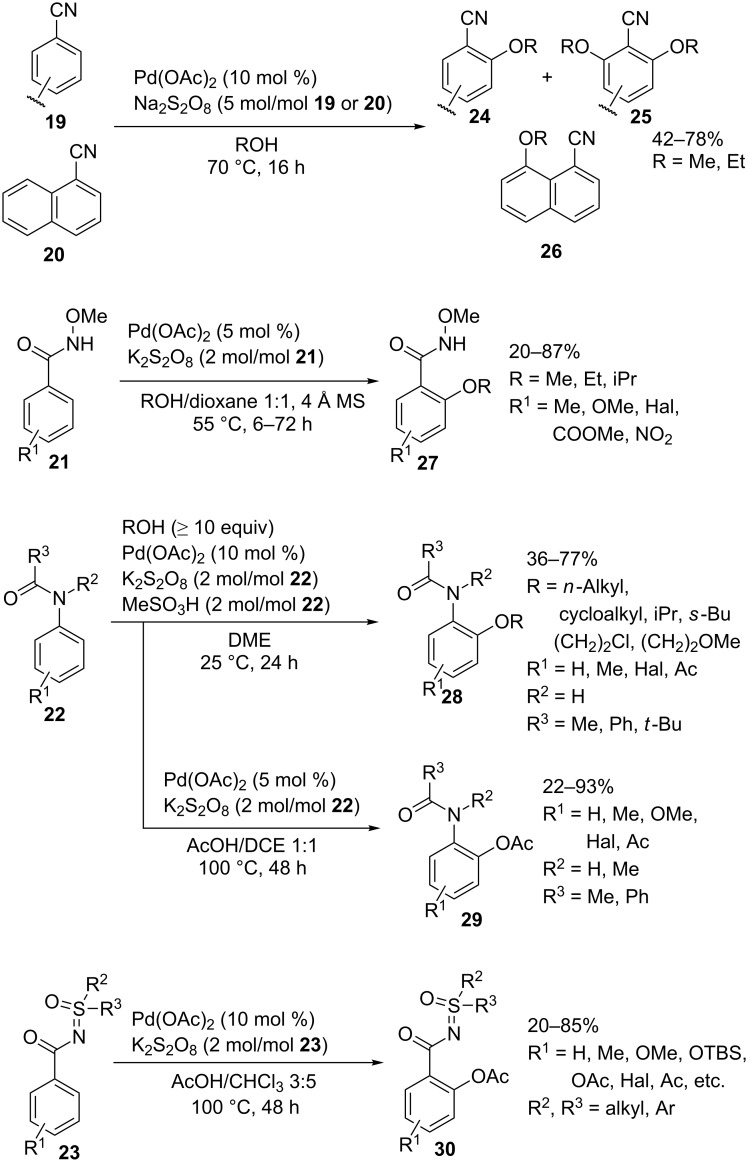
Pd(OAc)_2_/persulfate oxidative system in the *ortho*-alkoxylation and acetoxylation of arenes with nitrile, amide, and sulfoximine directing groups.

The Pd(OAc)_2_/persulfate system was used in the *ortho*-alkoxylation of arylnitriles **19**–**20** [48], *N*-methoxybenzamides **21** [49], and acetanilides **22** [50] and in the *ortho*-acetoxylation of acetanilides **22** [51] and sulfoximines **23** [52] to prepare cross-dehydrogenative C–O coupling products **24**–**30** (Scheme 6). The alkoxylation of 1-naphthonitrile **20** occurs not at the *ortho*-position but at the 8-position of the aromatic system to give product **26**. The acetoxylation takes place under more severe conditions compared with the alkoxylation. The acetoxylation employing the *S*-methyl-*S*-2-pyridylsulfoximine moiety as the bidentate directing group can be performed at lower temperature (50 °C instead of 100 °C, as in the case of CH-reagents **22** and **23**) [53].

**Scheme 7:**
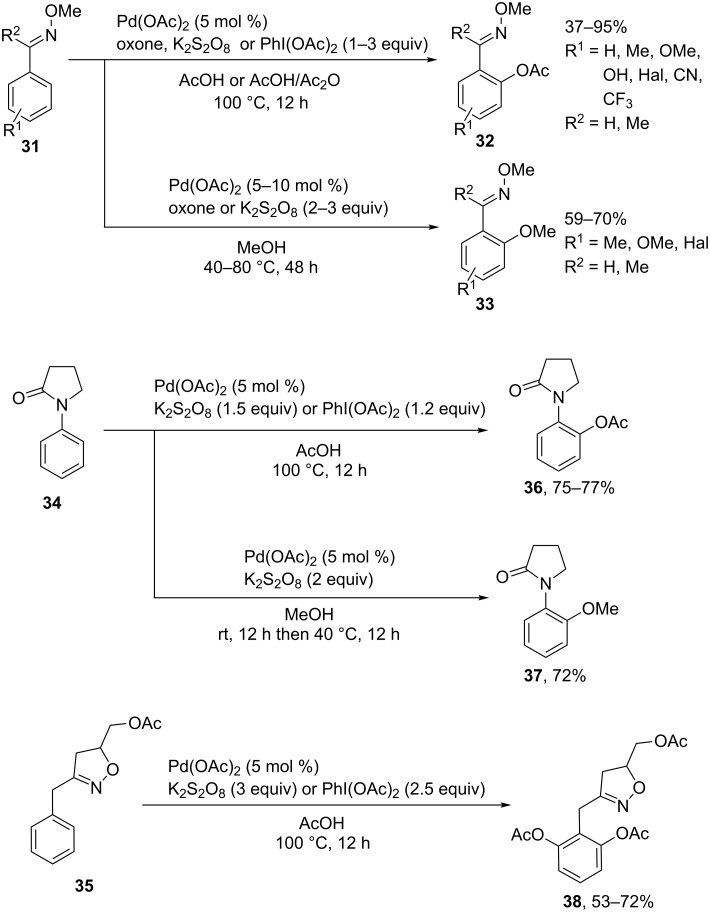
*ortho*-Acetoxylation and methoxylation of *O*-methyl aryl oximes, *N*-phenylpyrrolidin-2-one, and (3-benzyl-4,5-dihydroisoxazol-5-yl)methyl acetate.

The *ortho*-acetoxylation and methoxylation of *O*-methyl aryl oximes **31** with Pd(OAc)_2_ combined with such oxidants as oxone, potassium persulfate, and (diacetoxyiodo)benzene (Scheme 7, coupling products **32** and **33**) occur under similar conditions [54]. *N*-Phenylpyrrolidin-2-one (**34**) and (3-benzyl-4,5-dihydroisoxazol-5-yl)methyl acetate (**35**) react in a similar fashion to afford products **36–38**. Related *ortho*-acetoxylation reactions of the aryl group of methoxyimino-2-aryl acetates [55], 2-methoxyimino-2-arylacetamides [55], and *O*-acetyl aryl oximes [56] in the presence of the Pd(OAc)_2_/PhI(OAc)_2_ system were described.

**Scheme 8:**
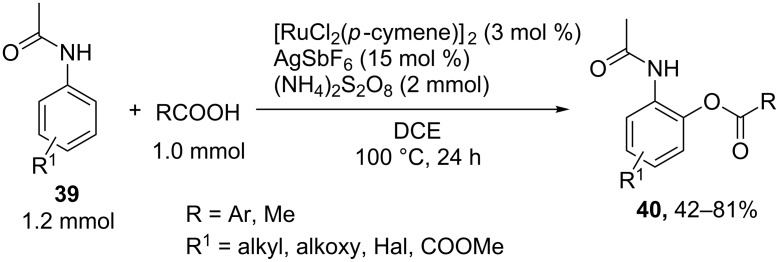
Ruthenium-catalyzed *ortho*-acyloxylation of acetanilides.

The ruthenium-catalyzed *ortho*-acyloxylation of acetanilides **39** with carboxylic acids in the presence of AgSbF_6_ and ammonium persulfate afforded products **40** (Scheme 8) [57]. This method can be used for the selective replacement of one of the two hydrogen atoms in the *ortho*-position of acetanilide; the molar ratio of the C- and O-reagents is close to stoichiometric.

**Scheme 9:**
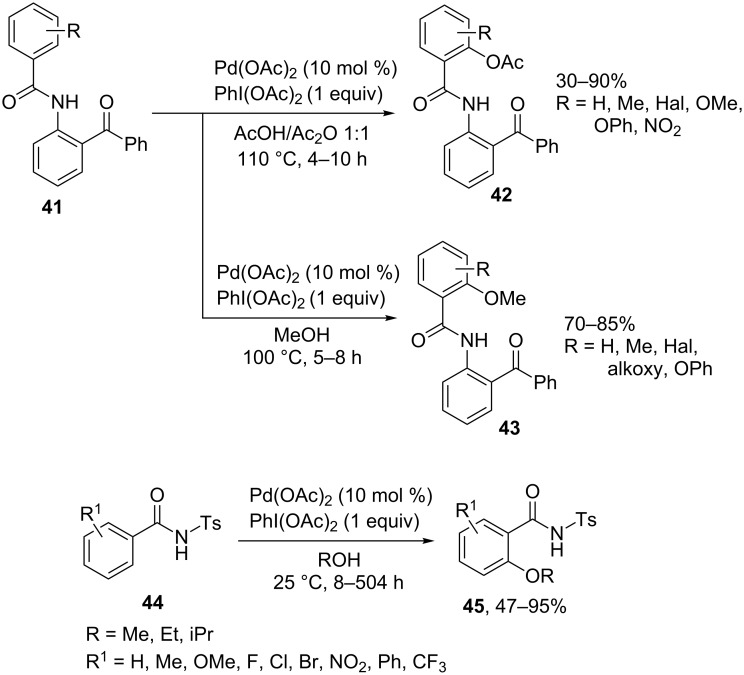
Acetoxylation and alkoxylation of arenes with amide directing group using Pd(OAc)_2_/PhI(OAc)_2_ oxidative system.

The acetoxylation (product **42**) and methoxylation (product **43**) of *N*-(2-benzoylphenyl)benzamides **41** at the *ortho*-position of the benzamide moiety of the substrate were performed using Pd(OAc)_2_ combined with PhI(OAc)_2_ as the oxidant (Scheme 9) [58]. The alkoxylation of *N*-tosylbenzamides **44** in the presence of the same oxidative system takes place at room temperature and gives products **45** [59]. The reactions of benzamides containing the nitro group at the *ortho*-position are most difficult to perform [58–59].

**Scheme 10:**
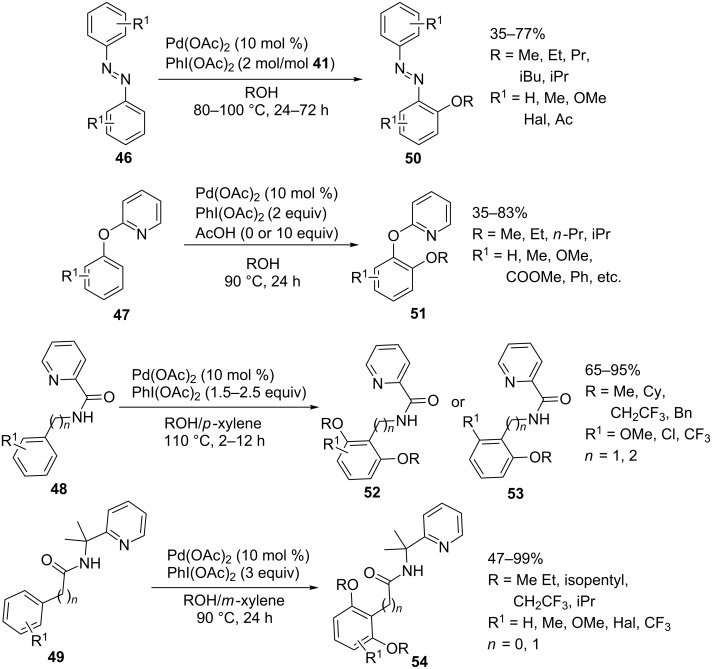
Alkoxylation of azoarenes, 2-aryloxypyridines, picolinamides, and *N*-(1-methyl-1-(pyridin-2-yl)ethyl)amides using the Pd(OAc)_2_/PhI(OAc)_2_ oxidative system.

The Pd(OAc)_2_/PhI(OAc)_2_ system was also employed to accomplish the *ortho*-alkoxylation of azoarenes **46** [60], 2-aryloxypyridines **47** [61], picolinamides **48** [62], and *N*-(1-methyl-1-(pyridin-2-yl)ethyl)amides **49** [63], resulting in the formation of products **50**–**54** (Scheme 10).

**Scheme 11:**
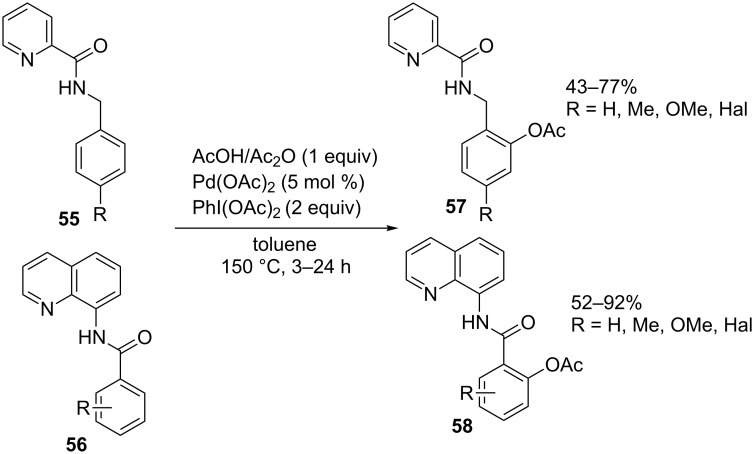
Acetoxylation of compounds containing picolinamide and quinoline-8-amine moieties using the Pd(OAc)_2_/PhI(OAc)_2_ system.

The *ortho*-acetoxylation of compounds containing picolinamide and quinoline-8-amine moieties (**55** and **56**, respectively) with the Pd(OAc)_2_/PhI(OAc)_2_ system in a AcOH/Ac_2_O mixture, resulting in the formation of products **57**, **58**, was performed at higher temperature (150 °C, Scheme 11) compared with the alkoxylation of structures **46**–**49** [64]. Under similar conditions, aryl phosphates and benzyl phosphonic monoacids were subjected to *ortho*-acetoxylation in the presence of the Pd(OAc)_2_/PhI(OAc)_2_ system; (diacetoxyiodo)benzene served as the source of the acetoxy group [65].

**Scheme 12:**
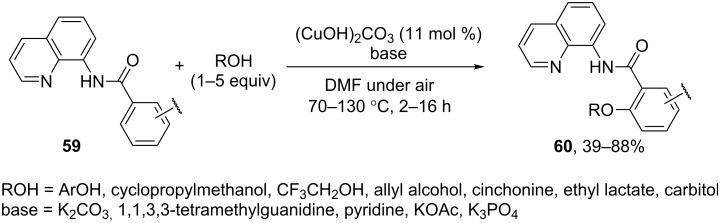
(CuOH)_2_CO_3_ catalyzed oxidative *ortho*-etherification using air as oxidant.

The quinoline-8-amine directing moiety was used for copper-catalyzed aerobic *ortho*-aryloxylation and alkoxylation of arenes **59** with electron donating or electron withdrawing substituents to afford products **60** [66] (Scheme 12). The method is applicable to a wide range of OH-reagents; the molar ratio OH-reagent/CH-reagent was 1:1 for aromatic OH-reagents and 5:1 for aliphatic alcohols. Different organic and inorganic bases were used depending on the structures of substrates.

**Scheme 13:**
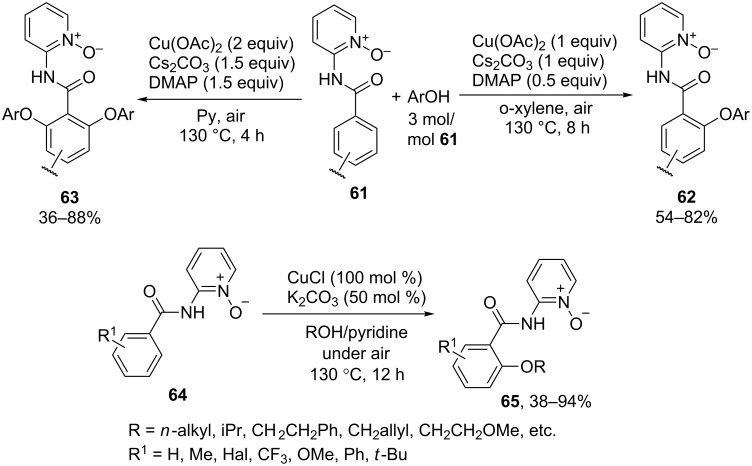
Copper-catalyzed aerobic alkoxylation and aryloxylation of arenes containing pyridine-N-oxide moiety.

The cross-dehydrogenative coupling of phenols and arenes **61** with a directing group containing a pyridine *N*-oxide moiety occurs in the presence of Cu(OAc)_2_ under air atmosphere to form coupling products with one (product **62**) or two (product **63**) equivalents of phenol (Scheme 13) [67]. The same directing group was used for aerobic *ortho*-alkoxylation of arenes **64** in the presence of CuCl and K_2_CO_3_ to afford monoalkoxylated products **65** [68].

**Scheme 14:**
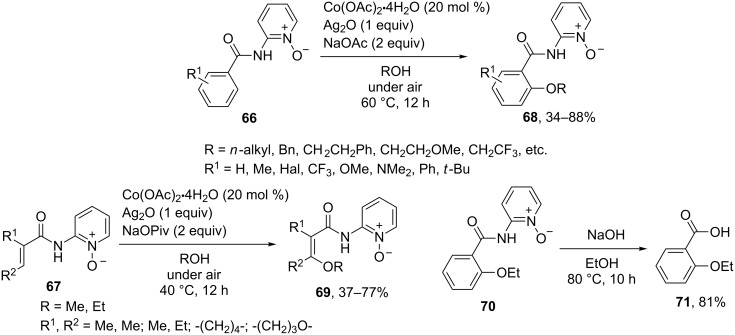
Cobalt-catalyzed aerobic alkoxylation of arenes and alkenes containing pyridine *N*-oxide moiety.

The 2-aminopyridine-1-oxide directing group was used in a rare example of a cobalt-catalyzed oxidative alkoxylation of arenes **66** and alkenes **67** to afford products **68** and **69** under mild contitions [69] (Scheme 14). The directing group can be removed to obtain the corresponding benzoic acid **71** from the cross-dehydrogenative coupling product **70** [69].

**Scheme 15:**
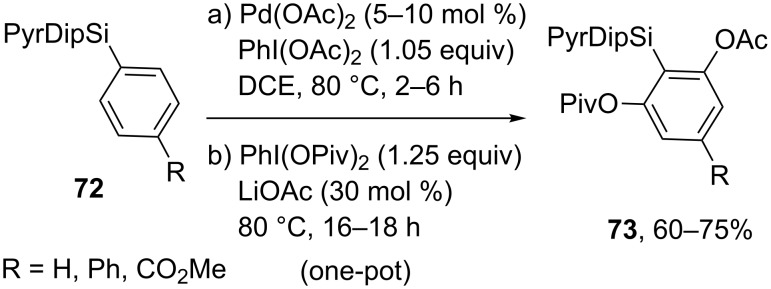
Non-symmetric double-fold C–H *ortho*-acyloxylation.

The majority of above mentioned directing groups cannot be easily removed or modified, thus limiting the scope of possible target products. To overcome these limitations, 2-pyridyldiisopropylsilyl (PyDipSi) [70–71] and 2-pyrimidyldiisopropylsilyl (PyrDipSi) [72–73] directing groups were proposed by the research group of Prof. V. Gevorgyan. The PyDipSi group was used for selective monoacetoxylation or pivaloyloxylation of the arene *ortho*-position [70–71], whereas the PyDipSi group allowed to achieve one-pot sequential acyloxylation of both *ortho*-positions affording, in particular, orthogonally protected resorcinol derivatives **73** in one step starting from PyrDipSi arenes **72** [72] (Scheme 15).

Pd(OAc)_2_ was used as catalyst with addition of AgOAc or LiOAc; PhI(OAc)_2_ or PhI(OPiv)_2_ were used as oxidants and sources of OAc and OPiv fragments, respectively, the syntheses were performed in dichloroethane at 80 °C for 1–168 h. After *ortho*-acyloxylation step silyl directing group can be removed or substituted by a desirable moiety (-Aryl, -OH, -B(pin), -I, etc.).

**Scheme 16:**
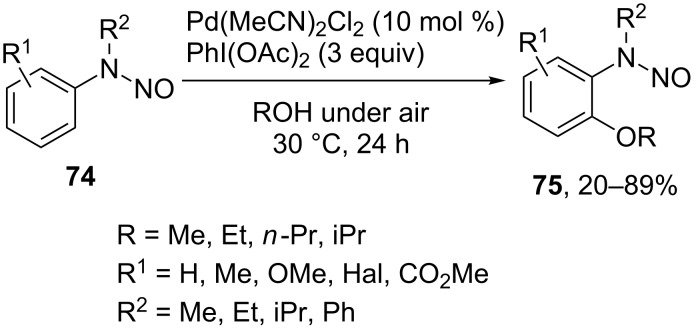
*N*-nitroso directed *ortho*-alkoxylation of arenes.

Another example of an easily modifiable directing group is an N-nitroso moiety that can be easily reduced to amine employing Fe/NH_4_Cl [74]. *N*-Nitroso directed C–H alkoxylation of arenes **74** was realized using the Pd(MeCN)_2_Cl_2_/PhI(OAc)_2_ oxidative system to obtain products **75** [74] (Scheme 16).

#### 1.2 Reactions involving C(sp^3^)*-*H bonds of C-reagents with alkyl groups

**Scheme 17:**
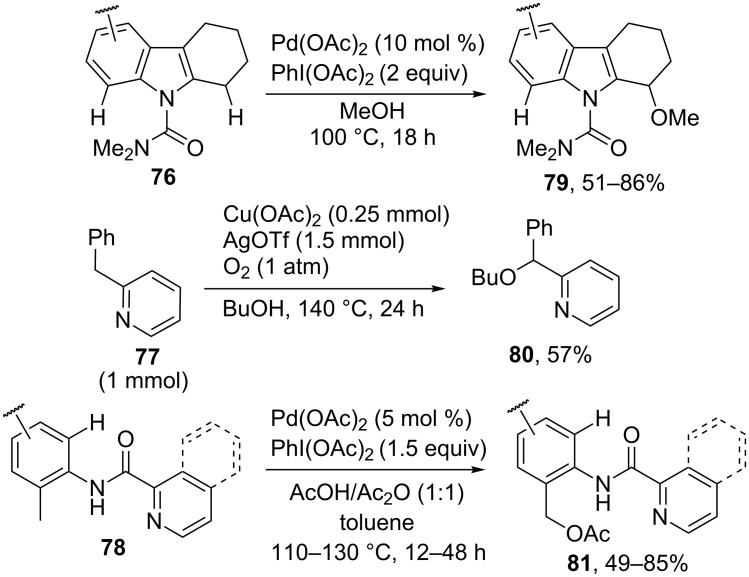
Selective alkoxylation and acetoxylation of alkyl groups.

In some studies, directing groups were used to accomplish the C–O coupling involving sp^3^-carbon atoms of C-reagents. Scheme 17 presents the alkoxylation of CH-reagents **76**, **77** and the acetoxylation of CH-reagents **78**. In these examples, the reaction occurs at the methylene group of the molecule (with the sp^3^-carbon atom) rather than at the *ortho*-position of the aromatic system, which is also adjacent to the directing group. The Pd(OAc)_2_/PhI(OAc)_2_ oxidative system was employed in the methoxylation of dimethylcarbamoyltetrahydrocarbazoles **76** [75] and the acetoxylation of compounds containing the picolinamide directing group **78** [76–77]; the butoxylation of the methylene group of 2-benzylpyridine **77** took place in the presence of the Cu(OAc)_2_/AgOTf/O_2_ system [47]. Cross-dehydrogenative C–O coupling products **79**–**81** were obtained, despite the potential possibility of the more profound oxidation of the methylene group to the keto group.

**Scheme 18:**
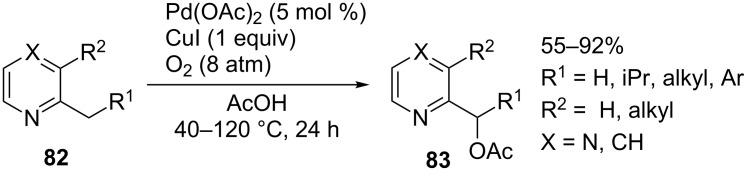
Acetoxylation of 2-alkylpyridines and related compounds.

The benzylic position of 2-alkylpyridines and related compounds **82** was acetoxylated using the Pd(OAc)_2_/CuI catalytic system in acetic acid under an oxygen pressure of 8 atm to prepare products **83** (Scheme 18) [78].

The acyloxylation of methyl or methylene groups of 8-methylquinoline and its derivatives in the presence of the Pd(OAc)_2_/PhI(OAc)_2_ [79] and Pd(OAc)_2_/ligand/O_2_ [80] oxidative systems was also described.

In the above considered examples, the oxidative C–O coupling occurs with the involvement of methyl or methylene groups directly bonded to the aromatic ring. The C–O coupling reactions involving unactivated alkyl groups of C-reagents are considered below.

**Scheme 19:**
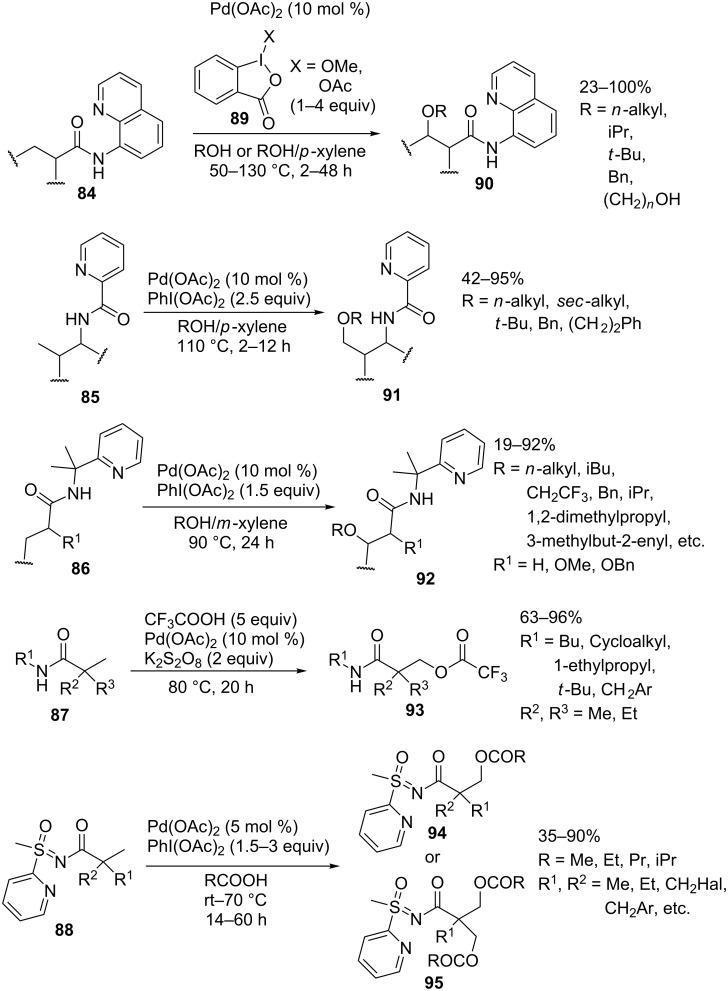
Acyloxylation and alkoxylation of alkyl fragments of substrates containing amide or sulfoximine directing groups.

The amide moiety was most commonly employed as the directing group. The alkoxylation of alkyl groups of *N*-(quinolin-8-yl)amides **84** [81], picolinamides **85** [62], and *N*-(2-pyridin-2-yl)propan-2-yl)amides **86** [63], the trifluoroacetoxylation of amides **87** [82], and the acyloxylation of compounds containing the *S*-methyl-*S*-pyridylsulfoximine moiety **88** [83] were accomplished to prepare coupling products **90**–**95** (Scheme 19). In some cases, the latter reaction proceeds efficiently even at room temperature. Iodine(III) compounds **89**, (diacetoxyiodo)benzene, or potassium persulfate served as oxidants. The former three reactions are applicable to a broad range of substrates and alcohols. The drawback is that a large excess of alcohols is required. Unlike the alkoxylation of **84**–**86**, the trifluoroacetoxylation of **87** was performed with structurally simple amides. This method is applicable only to α-disubstituted amides. The reactions with amides containing hydrogen in the α-position give coupling products **93** in substantially lower yields [82]. Similar limitations are encountered when performing the acyloxylation of *S*-methyl-*S*-pyridylsulfoximines **88**.

**Scheme 20:**
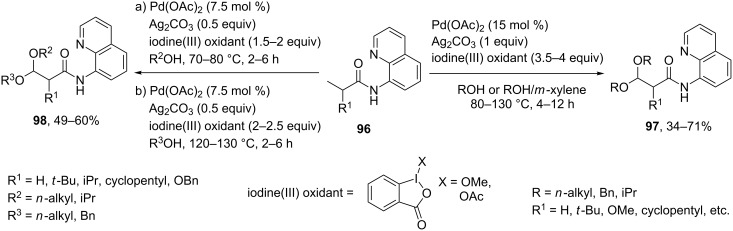
Palladium-catalyzed double sp^3^ C–H alkoxylation of *N*-(quinolin-8-yl)amides for the synthesis of symmetric and unsymmetric acetals.

Using the *N*-(quinolin-8-yl)amide directing group Pd(OAc)_2_ catalyzed the double sp^3^ C–H alkoxylation of **96** for the synthesis of symmetric acetals **97** and unsymmetric acetals [84] (Scheme 20). The method demonstrates good functional group tolerance and the applicability in the synthesis of products containing α-hydrogen next to an amide moiety.

**Scheme 21:**
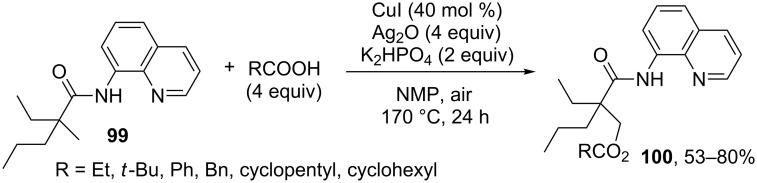
Copper-catalyzed acyloxylation of methyl groups of *N*-(quinolin-8-yl)amides.

Copper-catalyzed acyloxylation of methyl groups of *N*-(quinolin-8-yl)amides **99** was achieved [85] (Scheme 21, product **100**); the reaction requires higher temperatures (170 °C) then Pd(OAc)_2_ catalyzed alkoxylation of analogous substrates in the presence of iodine(III) oxidants (50–130 °C, Scheme 19 and Scheme 20). Under similar conditions methyl groups of *N*-(quinolin-8-yl)amides were acetoxylated using the Cu(OAc)_2_ catalyst (50 mol %) and AgOAc (3 equiv) as acetoxylating agent.

**Scheme 22:**
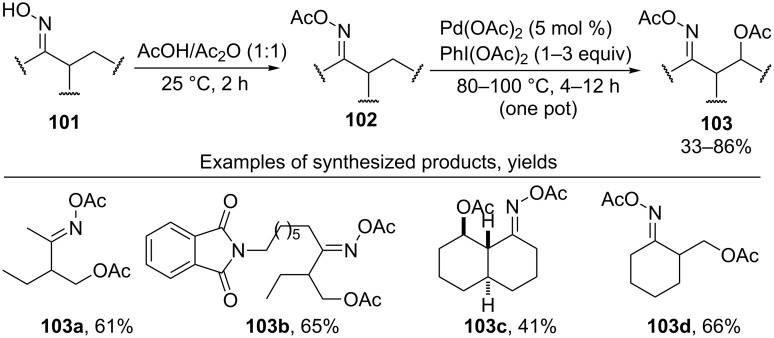
One-pot acylation and sp^3^ C–H acetoxylation of oximes.

The acetoxylation of alkyl groups of *O*-acetyl oximes **102** taking place in the presence of the Pd(OAc)_2_/PhI(OAc)_2_ system in a AcOH/Ac_2_O mixture affords products **103** (Scheme 22) [56]. The acylation of oxime **101** and the C–H acetoxylation of **102** are performed as a one-pot operation. In the acetoxylation of alkyl groups, the methyl group is more reactive than the methylene one.

The *O*-methyl oxime [54,86] or pyridine moieties [86] were employed as directing groups in the acetoxylation of alkyl groups using the Pd(OAc)_2_/PhI(OAc)_2_ system. The synthesis was carried out at 80–100 °C during a period of time from 5 min to 12 h in acetic acid, a 1:1 AcOH/Ac_2_O mixture, or dichloromethane. More recently, a Pd(OAc)_2_/NaNO_3_/O_2_ catalytic system was developed for acetoxylation of unactivated sp^3^-C–H bonds using the same directing groups [87]. Air or oxygen (1 atm) played a role of terminal oxidant and sodium nitrate was a redox co-catalyst; the reactions were performed in a AcOH/Ac_2_O solvent mixture at 100–110 °C for 18 h.

The oxazole moiety also acts as the directing group in the acetoxylation of alkyl groups with Pd(OAc)_2_/AcOO*t-*Bu or Pd(OAc)_2_/lauroyl peroxide oxidative systems; in these reactions acetic anhydride served as the source of the acetoxy groups [88]. Recently, Cu(OAc)_2_-mediated аcetoxylation of unactivated methyl fragments using the *N*-(quinolin-8-yl)amide directing group and AgOAc as oxidant was achieved without a Pd catalyst [89].

### 2 Aldehydes and alcohols as C-reagents in cross-dehydrogenative C–O coupling

There are numerous reactions, in which aldehydes are involved in the oxidative C–O coupling with alcohols under the action of oxidizing agents to form esters. In some cases, primary alcohols are used instead of aldehydes. It is commonly assumed that, under these reaction conditions, primary alcohols are oxidized to aldehydes followed by cross-dehydrogenative C–O coupling. These processes with aldehydes or primary alcohols as C-reagents giving esters are often referred to as oxidative esterification.

#### 2.1 Transition metal salt-catalyzed reactions using compounds with C=C, C=O, and C–Hal bonds as oxidants

One of the types of the oxidative esterification is based on the hydrogen transfer catalyzed by transition metal complexes. In these reactions, compounds with a double bond or a C–Hal bond act as oxidants (hydrogen acceptors). Examples of these reactions with aldehydes or primary alcohols as C–H reagents are given in Table 2.

**Table 2:** Transition metal salt-catalyzed oxidative coupling of primary alcohols or aldehydes with alcohols using compounds with C=C, C=O, and C–Hal bonds as oxidants.

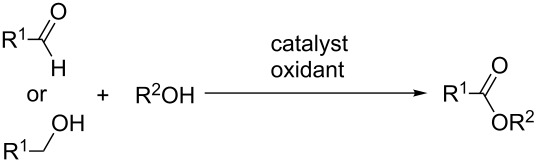					

CH-reagent	R^2^ (amount of R^2^OH)	Catalyst	Oxidant	Conditions; yields	Ref.

R^1^CH=O, R^1^CH_2_OH	Me (excess)	RuH_2_(CO)(PPh_3_)_3_/4,5-bis(diphenylphosphino)-9,9-dimethylxanthene	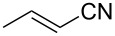	MeOH/PhMe (1:1), 4–48 h, 110 °C; 74–95%	[90–91]
R^1^CH_2_OH	Me (excess)	[CpIrCl_2_]_2_/2-methylaminoethanol/ Cs_2_CO_3_	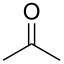	acetone, 24 h, room temperature; 23–92%	[92]
R^1^CH=O	Me, Et, iPr, *s*-Bu, CH_2_CF_3_ (excess)	Pd(OAc)_2_/2-dicyclohexylphosphino-2',4',6'-triisopropylbiphenyl/K_2_CO_3_	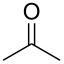	acetone/R^2^OH, 2–24 h, room temperature–50 °C; 7–99%	[93]
R^1^CH=O	Et (excess)	Pd(PPh_3_)_4_/K_2_CO_3_	BnCl or BnBr	EtOH, MW, 30 min, 90 °C; 65–93%	[94]
R^1^CH=O	*n*-alkyl, *sec-*alkyl, Bn, etc. (1 equiv)	PdCl_2_(PPh_3_)_2_/K_2_CO_3_	BnCl	THF, 20 h, 50 °C; 72–99%	[95]

Crotononitrile, acetone, and benzyl halides were used as oxidants. Ruthenium, iridium, and palladium complexes acted as catalysts. In most cases, structurally simple alcohols, which are taken in a large excess relative to the CH-reagent, served as OH-reagents. The exception is a study [95], in which the coupling was accomplished using an equivalent amount of alcohol.

#### 2.2 Oxidative systems based on noble metals and oxygen

The oxidative coupling of benzyl alcohols with aliphatic alcohols in the presence of Pd(II) salt/Ag(I) salt/base/oxygen system was proposed [96–97]. In the study [97], phosphine ligands were additionally employed. It is suggested that the coupling occurs through oxidation of benzyl alcohol to benzaldehyde. Alcohols (OH-reagents) are taken in a twofold molar excess [96] or are used as solvents [96–97]; the reaction time is 20–40 h at 45–80 °C. The aerobic oxidative coupling of aldehydes or primary alcohols as CH-reagents with low-molecular-weight alcohols was performed in the presence of heterogeneous catalysts, such as Au/TiO_2_ [98–100], Au/β-Ga_2_O_3_ [101], Au/polymer [102], and AuNiO_x_/SiO_2_-Al_2_O_3_-MgO [103]. In all the above-mentioned processes in the presence of heterogeneous catalysts, low-molecular-weight alcohols are used as the solvents or are taken in a large excess relative to the CH-reagent.

#### 2.3 Reactions catalyzed by N-heterocyclic carbenes

**Scheme 23:**
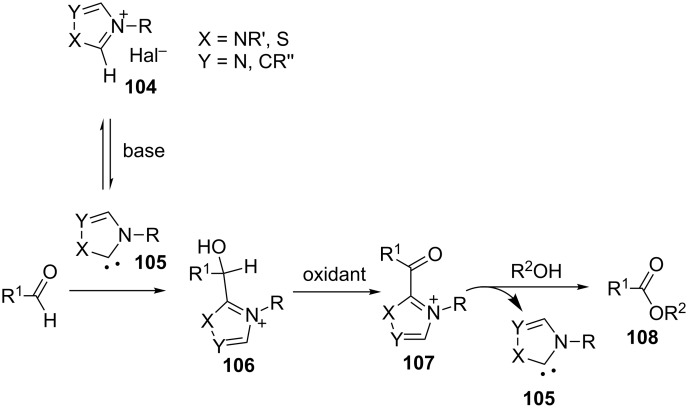
Possible mechanism of oxidative esterification catalyzed by N-heterocyclic nucleophilic carbene.

N-heterocyclic nucleophilic carbenes **105** have found use for the oxidative esterification. Scheme 23 shows, in a simplified way, the proposed mechanism of this type of cross-dehydrogenative C–O coupling [104–113]. The aldehyde is subjected to the attack of N-heterocyclic nucleophilic carbene **105**, which can be generated from the corresponding azolium salt **104**. The resulting intermediate **106** is oxidized to **107** followed by the nucleophilic attack by the alcohol to form ester **108**.

The mechanism of the aerobic oxidative coupling of benzaldehyde with methanol in the presence of the 4-ethyl-1-methyl-1*H*-1,2,4-triazolium iodide/DBU system was studied in detail [114]. It was shown that the reaction proceeds via another mechanism, involving the formation of 2-hydroxy-1,2-diphenylethanone from benzaldehyde followed by the oxidation of this intermediate to 1,2-diphenylethanedione.

**Scheme 24:**
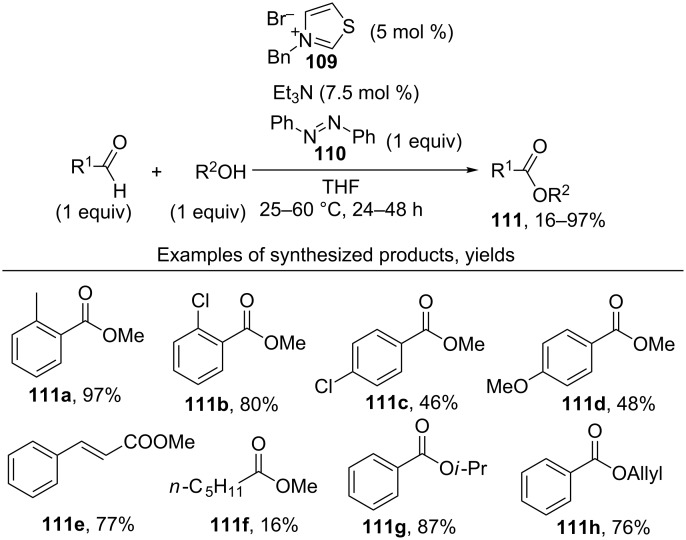
Oxidative esterification employing stoichiometric amounts of aldehydes and alcohols.

Scheme 24 shows an example of the oxidative coupling of aldehydes and alcohols, in which CH- and OH-reagents are taken in equivalent amounts [104]. Thiazolium salt **109** combined with triethylamine acted as the catalyst, and azobenzene **110** served as the oxidizing agent. Esters **111** were prepared in 16–97% yield.

**Scheme 25:**
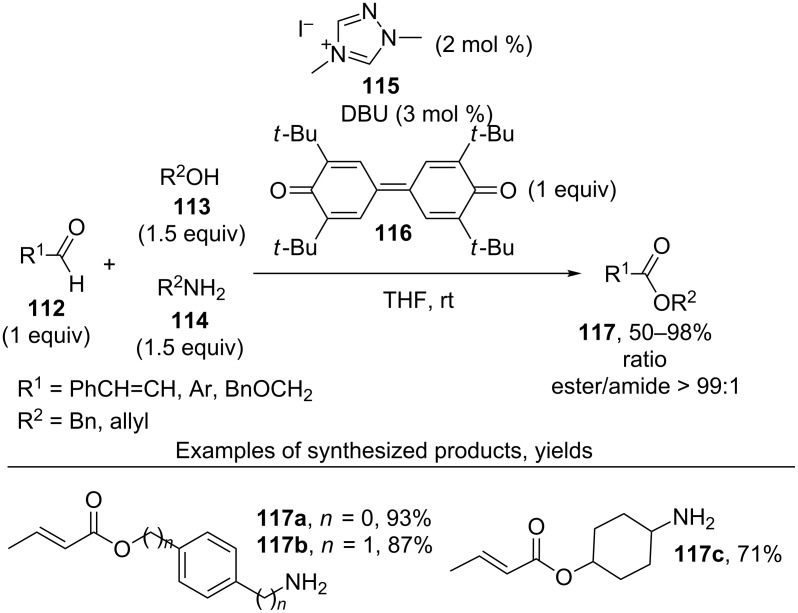
Selective oxidative coupling of aldehydes with alcohols in the presence of amines.

The selective oxidative coupling of aldehydes **112** and alcohols **113** in the presence of amines **114** using 1,4-dimethyltriazolium iodide (**115**), DBU and quinone **116** afforded esters **117**. This method was also applied to the synthesis of esters **117a**–**c** from amino alcohols (Scheme 25) [115–116].

Different conditions were proposed for the cross-dehydrogenative C–O coupling of aldehydes with alcohols and phenols catalyzed by N-heterocyclic carbenes (**118**, **129**) or their precursors, azolium salts (**119**–**128**), combined with bases (Table 3; the publications are summarized in chronological order). In most cases, alcohols were taken in an excess.

**Table 3:** Oxidative esterification of aldehydes with alcohols.

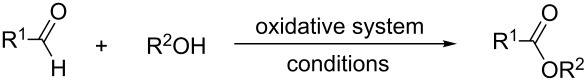				

R^1^	R^2^	Oxidative system	Conditions; yield	Ref.

Ar	*n-*alkyl, iPr	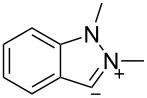 **118** (1 mol per mol of aldehyde), generated in situ	R^2^OH, bp; 17–75%	[117]
*n-*alkyl, *sec*-alkyl, *t-*Bu, etc.	Me, *n*-Pr, cyclohexyl, CH_2_CH_2_SiMe_3_, CH_2_CCl_3_, CH(Me)COOMe	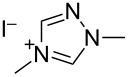 **119** (10 mol %) DBU (1.1 mol per mol of aldehyde) MnO_2_ (5 mol per mol of aldehyde)	R^2^OH (5 mol per mol of aldehyde) CH_2_Cl_2_, room temperature; 56–99%	[105–106]
Ar	Me, iPr, Ph, etc.	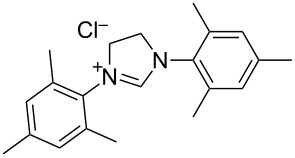 **120** Cs_2_CO_3_ (1.5 equiv), air	R^2^OH (3 equiv) cyclohexane, 25 °C, 10 h; 34–80%	[118]
Ar, PhCH=CH, cyclohexyl	Ar’	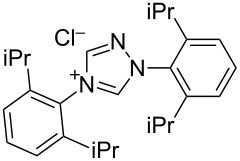 **121** (5 mol %) Pd(OAc)_2_ (5 mol %) Na_2_CO_3_ (4 equiv) air	aldehyde/phenol (3:2) xylene, 100 °C, 24–48 h; 25–99%	[119]
Ar, PhCH=CH, cyclohexyl	Ar’	**121** (20 mol %) Fe(OTf)_2_(20 mol %) *t-*BuOK (1 equiv) air	aldehyde/phenol (1:1) dioxane, 90 °C, 24 h; 15–89%	[107]
Ar, PhCH=CH, cyclohexyl	Me, Et, Bn, 2-PhEt, *s*-Bu, allyl, propargyl, 4-pentinyl, etc.	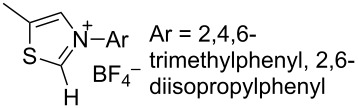 **122** (10 mol %) DBU (1 equiv) electrochemical oxidation, Bu_4_NBr (30 mol %)	aldehyde/alcohol (1:1.1) MeCN, room temperature; 60–97%	[108]
Ar	Me, Et, Pr, iPr	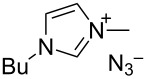 **123** (3 mol per mol of aldehyde)	aldehyde/alcohol (1:3) 50–60 °C; 50–90%	[120]
PhCH_2_CH_2_	Bn, allyl	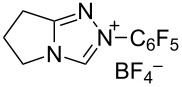 **124** (5 mol %) NHEt_2_ (1.1 equiv) Et_3_N (1.2 equiv) *N*-chlorosuccinimide (1 equiv)	aldehyde/alcohol (1:2) CH_2_Cl_2_, room temperature; 83–87%	[121]
Ar	Me, Et, iPr, allyl, propargyl, Bn	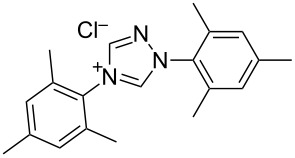 **125** (10 mol %) DBU (20 mol %) oxygen	aldehyde/alcohol (1:1.2) THF, 25 °C; 63–82%	[109]
Ar, PhCH=CH, cyclohexyl	Me, Et, *n*-Pr, Bn, allyl, propargyl	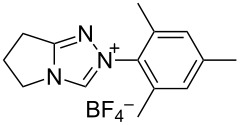 **126** (2.5 mol %) [Ru(2,2’-bipyrazine)_3_](PF_6_)_2_ (5 mol %), air	aldehyde/alcohol (1:10) MeCN, room temperature; 15–81%	[110]
Ar, PhCH_2_CH_2_	Me, Bn, allyl, CH_2_CCl_3_	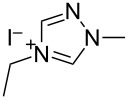 **127** (15 mol %) DBU (110 mol %) air	THF/ROH (1:1) or 3 equiv ROH in THF, room temperature; 15–94%	[114,122]
Ar, *n-*alkyl	*n-*alkyl, Bn, CH_2_CH_2_NEt_2_	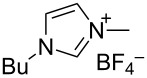 **128** (solvent) DBU (1 equiv), Cs_2_CO_3_ (3 equiv), MnO_2_ (3 equiv)	aldehyde:alcohol (1:3) 25 °C, 24 h; 25–91%	[111]
Ar, CH=CHPh, CH=CH(2-C_6_H_4_OMe)	Bn, CH=CHPh, Et, Ph, (CH_2_)_3_Ph	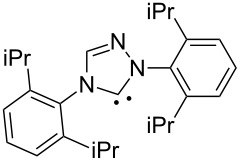 **129** (5 mol %) TEMPO (2 equiv)	aldehyde:alcohol (1:1.5) toluene, 100 °C, 4–6 h 60–86%	[112]

The oxidative esterification in the presence of azolium salts **119**, **121**, and **125** was performed with primary alcohols instead of aldehydes (Table 4). It is supposed that under the reaction conditions primary alcohol is initially oxidized to aldehyde without the participation of N-heterocyclic carbene [106,113,123–124].

**Table 4:** Oxidative esterification using primary alcohols as C-reagents.

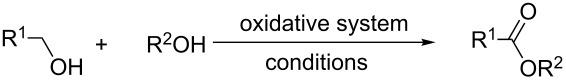				

R^1^	R^2^	Oxidative system	Conditions; yield	Ref.

substituted vinyl, alkynyl, Ar, Bn, etc.	Me, Bu, iPr, CH_2_CCl_3_, CH_2_CH_2_OMe, CH_2_CH_2_OTMS, etc.	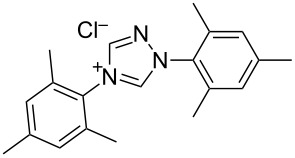 **125** (10–50 mol %), DBU (10–50 mol %), MnO_2_ (15 equiv)	R^2^OH or R^2^OH (5 equiv) in toluene, 23 °C; 65–95%	[113]
RCH=CH, PhCH=CMe, PhC≡C, Ar	Me, *n-*Bu, iPr	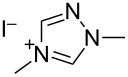 **119** (10 mol %), DBU (10–110 mol %), MnO_2_ (15 mol per mol of R^1^OH)	R^2^OH or R^2^OH (3–5 equiv) in toluene, 23 °C; 73–95%	[106]
ArCH_2_, allyl	Ar	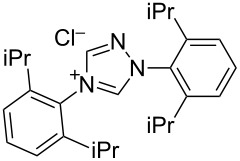 **121** (10 mol %), Pd(OAc)_2_ (5 mol %), Na_2_CO_3_ (0.5 mol per mol of phenol), oxygen	R^1^OH/R^2^OH (3:2) xylene, 130 °C, 36 h; 35–95%	[123]
ArCH_2_, allyl, Bu	Ar	**121** (10 mol %), [RuCl_2_(*p*-cymene)]_2_ (5 mol %), Cs_2_CO_3_ (10 mol %), oxygen	R^1^OH/R^2^OH (3:2) xylene, 130 °C, 24 h; 50–95%	[124]

The oxidative esterification of aldehydes with racemic mixtures of alcohols catalyzed by chiral N-heterocyclic carbenes was used for the kinetic separation of enantiomers of alcohols [125–126].

#### 2.4 Reactions using halogen-containing oxidative systems

This section considers cross-dehydrogenative C–O coupling reactions with aldehydes and primary alcohols as C-reagents in which halogens and their compounds, for example, molecular iodine, the Bu_4_NI/*t-*BuOOH system, organic iodine(III or V) compounds, bromides combined with oxidants, hypochlorite, and so on, acted as oxidants.

**Scheme 26:**
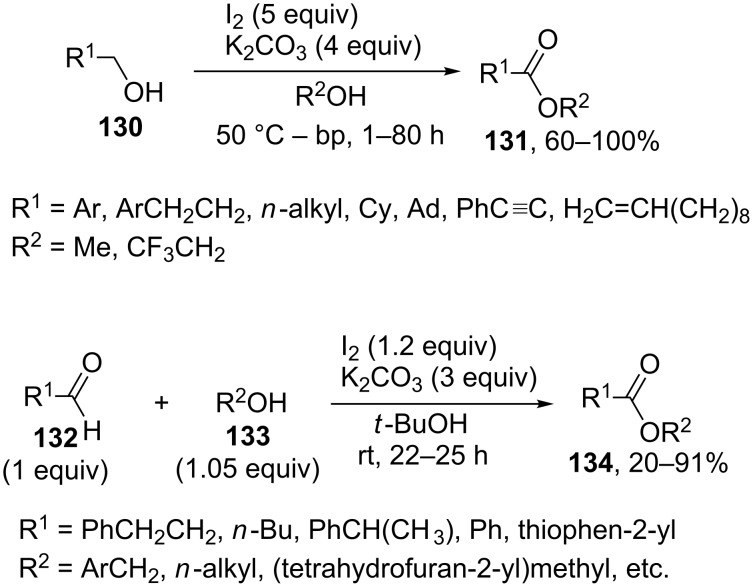
Iodine mediated oxidative esterification.

The oxidative coupling of primary alcohols **130** with methanol or trifluoroethanol and the oxidative coupling of aldehydes **132** with structurally diverse alcohols **133** were performed using molecular iodine in the presence of potassium carbonate (Scheme 26) [127]. In the former case, methanol or trifluoroethanol served as the solvent. In the latter case, the reaction was carried out in *tert*-butanol; the amount alcohol was nearly equivalent relative to aldehyde. It is suggested that the reaction proceeds through the formation of hemiacetal from alcohol and aldehyde, which is oxidized by iodine to ester **131** or **134**. In the coupling of two alcohols, one of them is initially oxidized to aldehyde by iodine.

In the oxidative coupling of aldehydes or primary alcohols, the second reagent (alcohol) usually served as the solvent. The oxidation was performed with halogen-containing reactants: I_2_/KOH [128], KI_cat_/*t-*BuOOH [129], I_2cat_/PhI(OAc)_2_ [130], I_2_/NaNO_2_ [131], NaBr/PhI(OAc)_2_ [132], LiBr/NaIO_4_/H_2_SO_4_ [133], Bu_4_NBr/NaOCl [134], NaOCl/AcOH [135], Py·HBr_3_ [136], *N*-bromosuccinimide/pyridine [137], *N*-iodosuccinimide/K_2_CO_3_ [138], and *N*,*N*'-diiodo-*N*,*N*'-1,2-ethanediylbis(*p*-toluenesulfonamide) [139].

**Scheme 27:**
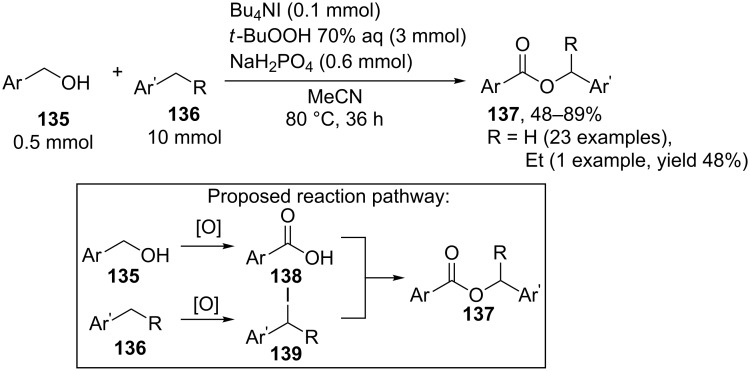
Oxidative C–O coupling of benzyl alcohols with methylarenes under the action of Bu_4_NI/*t-*BuOOH system in the presence of NaH_2_PO_4_.

The oxidative C–O coupling of benzyl alcohols **135** with alkylarenes **136** took place under the action of the Bu_4_NI/*t-*BuOOH system in the presence of NaH_2_PO_4_ [140]. It was proposed that under the reaction conditions benzyl alcohol **135** is oxidized to carboxylic acid **138**, while alkylarene gives iodide **139**; the nucleophilic substitution between the carboxylate anion and benzyl iodide affords coupling product **137** [140] (Scheme 27).

This mechanism differs from that proposed in the study [141]. According to the latter mechanism, the benzylic carbocation rather than benzyl iodide is generated and this carbocation undergoes nucleophilic attack by carboxylic acid. This mechanism is confirmed by the fact that iodide is inert under the reaction conditions. The conditions of the oxidative coupling used in the study [140] differ from the conditions described in the work [141]. However, it should be noted that the formation of benzyl iodide was not experimentally confirmed in the study [140], and the presence of this species was proposed based on the literature data.

**Scheme 28:**
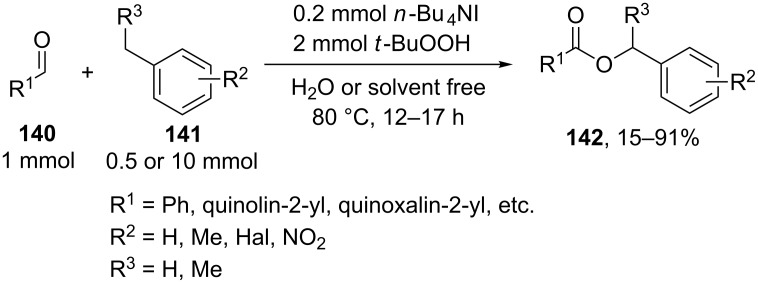
Oxidative coupling of methyl- and ethylarenes with aromatic aldehydes under the action of Bu_4_NI/*t-*BuOOH system.

The coupling of methyl- and ethylarenes **141** with aromatic aldehydes **140** was performed with the Bu_4_NI/*t-*BuOOH system in the presence of an excess of either alkylarene **141** or aldehyde **140** [142]. It was supposed that the coupling occurs through the generation of *tert*-butoxyl radicals, which abstract a hydrogen atom from the benzylic position of the C-reagent to form the C-radical, which is oxidized to the carbocation; in turn, the aldehyde is oxidized to acid, which reacts with the carbocation to give the target coupling product **142** (Scheme 28).

It was shown that under the reaction conditions, *tert*-butyl perester is generated as an important intermediate from aldehyde and *t-*BuOOH, and this intermediate can serve as the source of acid and *tert*-butoxyl radicals [142].

**Scheme 29:**
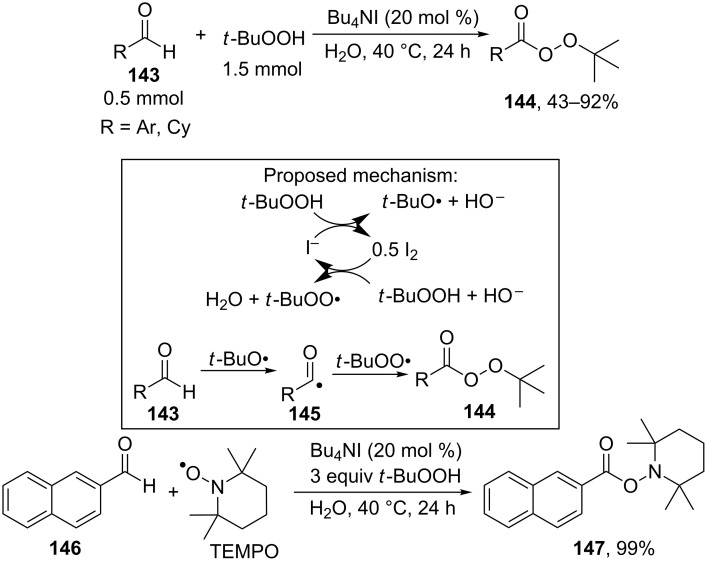
Cross-dehydrogenative C–O coupling of aldehydes with *t-*BuOOH in the presence of Bu_4_NI.

*tert*-Butyl peresters **144** were synthesized on a preparative scale by the cross-dehydrogenative C–O coupling of aldehydes **143** with *t-*BuOOH in the presence of Bu_4_NI [143]. It is proposed that *tert*-butyl peresters **144** are produced as a result of the recombination of acyl radicals **145** and *tert*-butyl peroxide radicals. The radical reaction mechanism was confirmed by the experiment, in which acyl radicals generated from aldehyde **146** were trapped by the stable radical TEMPO, and trapping product **147** was obtained in an almost quantitative yield (Scheme 29).

**Scheme 30:**
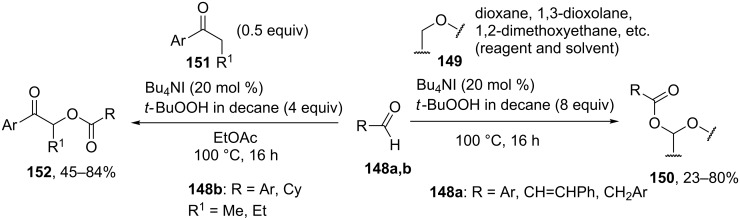
Bu_4_NI-catalyzed α-acyloxylation reaction of ethers and ketones with aldehydes and *t*-BuOOH.

The reaction of aldehydes **148a** with ethers **149** in the presence of Bu_4_NI and *t*-BuOOH generated corresponding α-acyloxy ethers **150**. Reactions between (hetero)aromatic aldehydes or cyclohexanecarbaldehyde **148b** with arylalkyl ketones **151** under similar conditions resulted in α-acyloxy ketones **152** [144] (Scheme 30). The plausible mechanism includes the formation of *tert*-butyl peresters from the aldehydes.

**Scheme 31:**
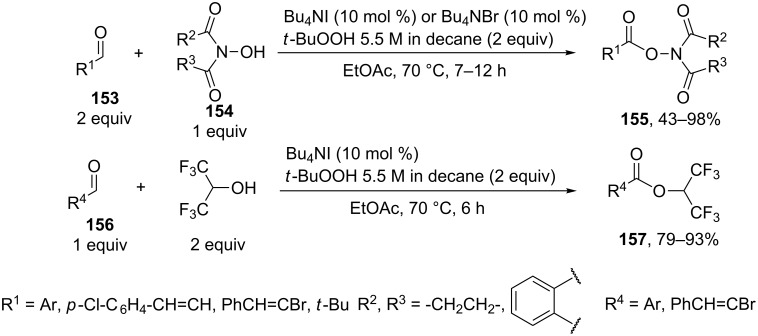
Oxidative coupling of aldehydes with *N*-hydroxyimides and hexafluoroisopropanol.

*N*-Hydroxyimides **154** are efficient OH-reagents in the coupling with aldehydes and primary alcohols. Their oxidative coupling products, so-called activated esters **155**, readily react with nucleophiles, alcohols or amines, due to which they are employed for the preparation of esters and amides. This type of coupling was accomplished using iodine-containing oxidants. The proposed reaction mechanism involves the nucleophilic addition of *N*-hydroxyimides to aldehydes followed by the oxidation of the resulting adduct to form the activated ester. For example, the C–O coupling of aldehydes **153** with *N*-hydroxyimides **154** was carried out in the presence of the Bu_4_NHal/*t-*BuOOH system (Hal = I or Br) [145]. This method is applicable to the oxidative coupling of aldehydes **156** with hexafluoroisopropanol giving esters **157**. One of the coupling components is added in a twofold excess (Scheme 31).

**Scheme 32:**
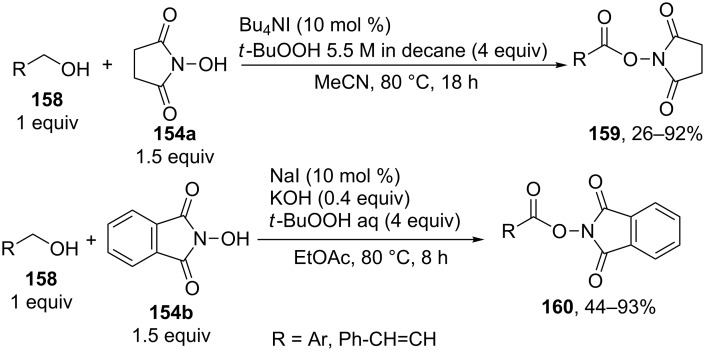
Oxidative coupling of alcohols with *N*-hydroxyimides.

A similar oxidative coupling reaction was performed with primary alcohols **158** as CH-reagents and *N*-hydroxyimides **154a**,**b** [146]. The coupling reaction with *N*-hydroxyphthalimide (**154b**) was carried out using the NaI/aqueous *t-*BuOOH/KOH system instead of Bu_4_NI/*t-*BuOOH in decane [146]. The resulting activated esters **159** and **160** were isolated or used in the one-pot reaction with amines to prepare amides (Scheme 32).

**Scheme 33:**
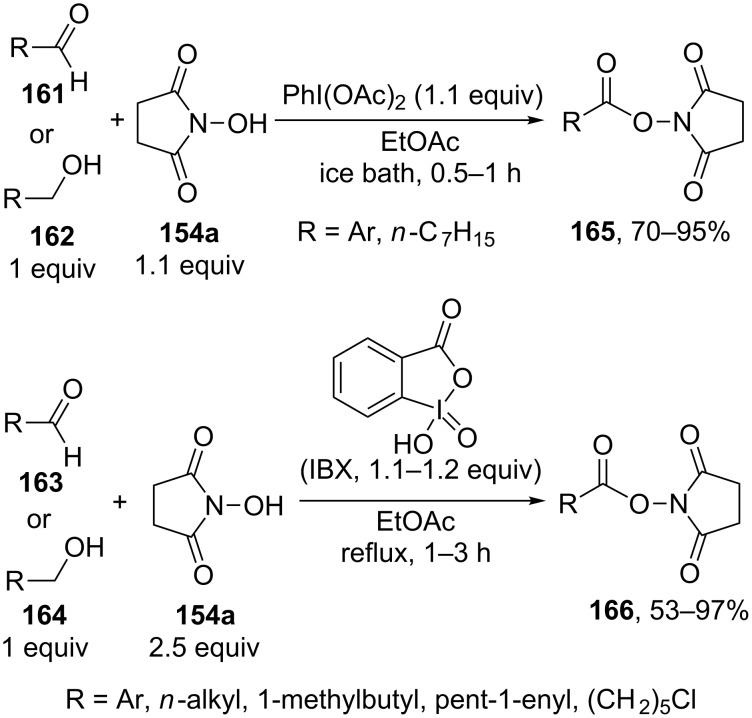
Oxidative coupling of aldehydes and primary alcohols with *N*-hydroxyimides using (diacetoxyiodo)benzene or iodoxybenzoic acid (IBX) as oxidants.

The oxidative C–O coupling of alcohols and aldehydes **161–164** with *N*-hydroxysuccinimide (**154a**) was performed in the presence of (diacetoxyiodo)benzene [147] or iodoxybenzoic acid (IBX) [148] as the oxidant. The authors hypothesized that the reaction proceeds via the nucleophilic addition of *N*-hydroxysuccinimide (**154a**) to aldehyde followed by the oxidation of the adduct that formed with iodine(III) [147] or iodine(V) [148] compounds to form products **165**, **166** (Scheme 33).

**Scheme 34:**
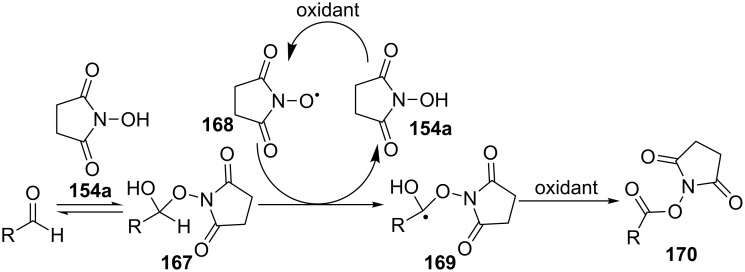
Proposed mechanism of the oxidative coupling of aldehydes and *N*-hydroxysuccinimide under action of (diacetoxyiodo)benzene.

In the study [149], activated esters were synthesized by the oxidative coupling of aldehydes and *N*-hydroxysuccinimide in the presence of (diacetoxyiodo)benzene, and these compounds were subjected, without isolation, in the reaction with amines to prepare amides. The reaction was performed at room temperature with *N*-hydroxysuccinimide (**154a**) taken in an equivalent amount or a small excess relative to aldehyde. Iodoxybenzoic acid (IBX) or the Co(OAc)_2_·4H_2_O/O_2_ system proved to be less efficient in the coupling reactions compared with (diacetoxyiodo)benzene. According to the proposed radical mechanism, *N*-hydroxysuccinimide (**154a**) adds to aldehyde to form intermediate **167** followed by the hydrogen atom abstraction from **167** by succinimide-*N*-oxyl radical **168** to give radical **169** and the oxidation of the latter resulted in the formation of coupling product **170** (Scheme 34). Radical intermediates **168** and **169** were detected by ESR spectroscopy [149].

**Scheme 35:**
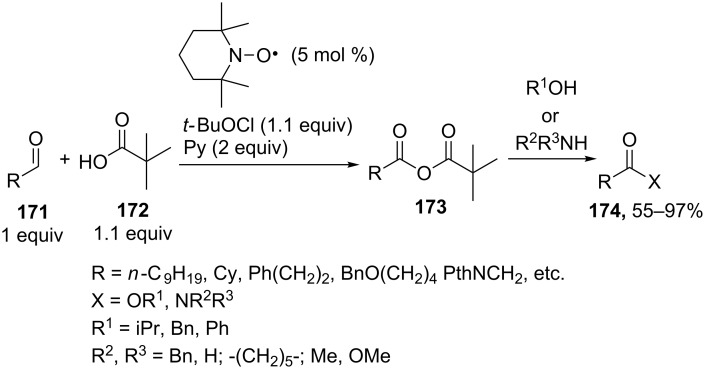
Oxidative coupling of aldehydes with pivalic acid (**172**).

The oxidative coupling of aldehydes **171** with pivalic acid (**172**) was performed using the TEMPO_cat_/*t-*BuOCl system; the coupling products, unsymmetrical anhydrides **173**, were employed in the synthesis of esters and amides **174** (Scheme 35) [150].

It is suggested that pivalic acid **172** adds to aldehyde **171** followed by the oxidation of the intermediate that formed with the oxoammonium salt, which is generated from TEMPO and *t-*BuOCl, to yield anhydride **173**.

#### 2.5 Oxidative systems based on transition metal salts and peroxides

**Scheme 36:**
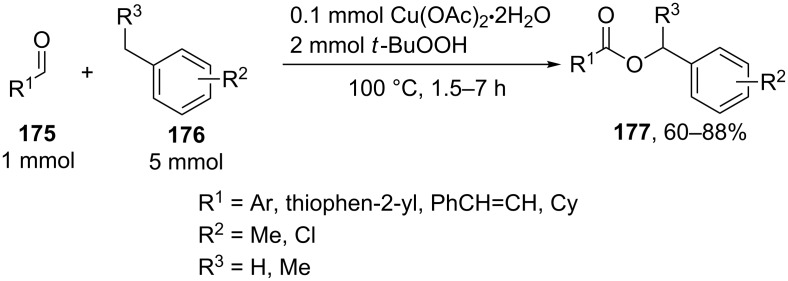
Oxidative C–O coupling of aldehydes with alkylarenes using the Cu(OAc)_2_/*t-*BuOOH system.

Esters **177** were synthesized by the oxidative C–O coupling of aldehydes **175** with alkylarenes **176** using the Cu(OAc)_2_/*t-*BuOOH system (Scheme 36) [151]. The coupling was performed with toluene, xylenes, 1,3,5-trimethylbenzene, 2,4-dichlorotoluene, and ethylbenzene.

**Scheme 37:**
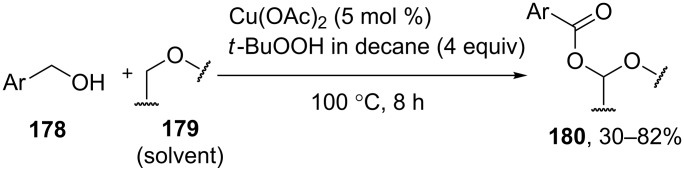
Copper-catalyzed acyloxylation of C(sp^3^)-H bond adjacent to oxygen in ethers using benzyl alcohols.

α-Acyloxy ethers **180** were synthesized by the oxidative coupling of benzyl alcohols **178** with ethers **179** (dioxane, tetrahydropyran, tetrahydrofuran, 1,2-dimethoxyethane) using Cu(OAc)_2_/*t*-BuOOH system [152] (Scheme 37).

In a series of works, the oxidative coupling of alcohols, aldehydes, or formamides with 1,3-dicarbonyl compounds or phenols was accomplished in the presence of *tert*-butyl hydroperoxide and copper salts (Table 5). In most cases, the range of phenols applicable to the coupling is limited to 2-acylphenols. However, 2-(benzothiazol-2-yl)phenol was used along with 2-acylphenols as the OH-reagent in the study [153].

**Table 5:** Oxidative coupling of alcohols, aldehydes, or formamides with 1,3-dicarbonyl compounds or phenols in the presence of *tert*-butyl hydroperoxide and copper salts.

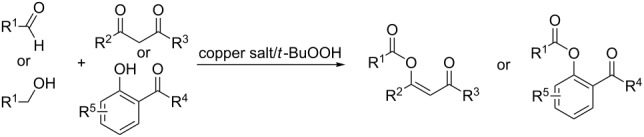			

C-reagent	O-reagent	Molar ratio C-reagent/O-reagent; conditions; yields	Ref.

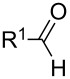 R^1^ = Ar, *n*-pentyl, cyclohexyl, diethylmethyl	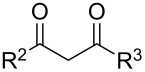 R^2^ = Me, Et, CH_2_Cl, Ph R^3^ = Me, Et, OMe, OEt	1:1.1; CuBr (2.5 mol %), *t-*BuOOH (5.5 M in decane, 1.5 equiv), 80 °C, 16 h; 57–89%	[154]
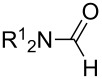 R^1^_2_ = di-Me, di-Et, di-iPr, -(CH_2_)_5_-	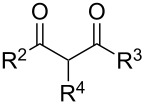 R^2^, R^4^ = alkyl, Ph, -(CH_2_)_4_-; R^4^ may be H; R^3^ = alkoxy, BnO 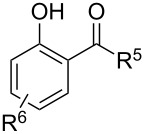 R^5^ = Me, OMe, Ph, NHPh R^6^ = H, OMe, Br	Formamide as the solvent; CuBr_2_ or Cu(OAc)_2_ (5 mol %), *t-*BuOOH (70% aq, 1.5 equiv), 80 °C, 3 h; 62–86%	[155]
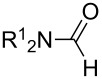 R^1^_2_ = di-Me, di-Et, di-iPr, -(CH_2_)_5_-	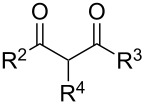 R^2^, R^4^ = alkyl, Ph, -(CH_2_)_4_-; R^4^ may be H; R^3^ = alkoxy, BnO 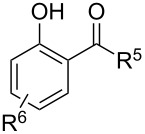 R^5^ = Me, OMe, Ph, NHPh R^6^ = H, OMe, Hal	Formamide as the solvent; CuCl (1 mol %), *t-*BuOOH (70% aq, 6 equiv), 70 °C, 15–30 min; 61–99%	[156]
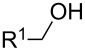 R^1^ = Ar, *n-*alkyl, cyclohexyl, CH_2_CH_2_Ph,	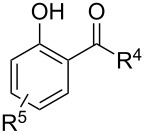 R^4^ = Me, OMe, OEt, OBn, Ph 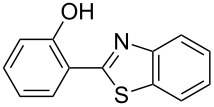	2:1; Cu(OAc)_2_ (5 mol %), *t-*BuOOH (70% aq, 4 equiv), DMSO, 80 °C, 20 h; 35–88%	[153]
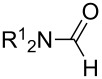 R^1^_2_ = di-Me, di-Et, di-iPr, -(CH_2_)_5_-, -(CH_2_)_2_O(CH_2_)_2_-	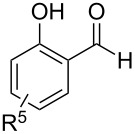 R^5^ = H, Me, *t-*Bu, OMe, NEt_2_, Cl, Br, etc*.*	Formamide as the solvent; CuCl (1–2 mol %), *t-*BuOOH (70% aq, 6 equiv), 80 °C, 15–90 min; 26–99%	[157]
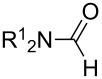 R^1^_2_ = di-Me, di-Et	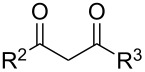 R^2^ = Me, Ph, *n-*Pr, *etc*. R^3^ = Me, OMe, OEt, Bn, allyl, etc.	Formamide as the solvent; CuO/α-Fe_2_O_3_/carbon nanotubes, *t-*BuOOH (70% aq, 1.5 equiv), 80 °C, 4 h; 40–80%	[158]
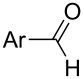	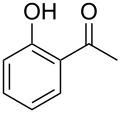	1:1; CuO/α-Fe_2_O_3_/carbon nanotubes, *t-*BuOOH (70% aq, 1.5 equiv), DMSO, 80 °C, 10 h; 20–82%	[158]

In the coupling with aldehydes, the components are taken in a nearly stoichiometric molar ratio. In the coupling with formamides, the latter served as the solvent. In the studies [155–156], 1,3-cyclohexanedione was used as the OH-reagent along with 1,3-ketoesters. However, the reaction with 1,3-cyclohexanedione gave products in low yields (19–26%). The coupling of a number of substituted salicylaldehydes with formamides was performed; the aldehyde group, which is prone to oxidation, remaining intact [157].

The coupling of aldehydes and formamides with 2-substituted phenols was carried out in the presence of heterogeneous catalysts, such as CuO on α-Fe_2_O_3_-modified carbon nanotubes (a magnetically separable catalyst) [158] and the metal-organic framework Cu_2_(4,4’-biphenyldicarboxylate)_2_(4,4’-bipyridine) [159].

Aldehydes were oxidized to esters in alcohols with V_2_O_5_/H_2_O_2aq_/HClO_4_ [160], V_2_O_5_/percarbonate, sodium perborate/HClO_4_ [161], VO(acac)_2_/H_2_O_2aq_ [162], Cu(ClO_4_)_2_/*t-*BuOOH in decane/InBr_3_ [163], Fe(ClO_4_)_3_/H_2_O_2aq_ [164], γ-Fe_2_O_3_-SiO_2_-supported heteropoly acids combined with H_2_O_2aq_ [165], silica gel-immobilized manganese phthalocyanine/H_2_O_2aq_ [166], a Ni(II) complex/H_2_O_2aq_ [167], and ZnBr_2_/H_2_O_2_ [168].

**Scheme 38:**
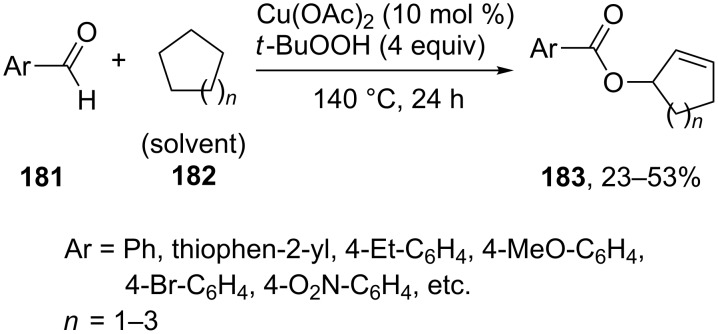
Oxidative C–O coupling of aromatic aldehydes with cycloalkanes.

The oxidative C–O coupling of aromatic aldehydes **181** with cycloalkanes **182** was accomplished in the presence of the Cu(OAc)_2_/*t-*BuOOH system to prepare products **183**. This reaction is unusual in that it involves the cleavage of four C–H bonds, including unactivated C–H bonds of cycloalkane, and the formation of two C–O bonds and one C=C double bond [169]. The yields of products **183** were not higher than 53%. However, the transformation is a rare example of the oxidative coupling with the participation of inert CH-reagents, cycloalkanes, to form a product, which can be subjected to higher oxidation under the conditions of oxidative coupling (Scheme 38).

Related oxidative C–O coupling reactions of alkanes with methylarenes and carboxylic acids are discussed in sections 4.1 and 6, respectively. Different radical mechanisms were proposed, the common feature is the generation of alkene via the radical dehydrogenation of alkane.

#### 2.6 Other oxidative systems

Esters were synthesized from aldehydes and methanol (6 equiv excess relative to aldehyde) using pyridinium dichromate in DMF [170]. Methyl esters were synthesized also from aromatic aldehydes, α,β-unsaturated aldehydes, or allylic alcohols in the presence of the DDQ (2,3-dichloro-5,6-dicyanobenzoquinone)/amberlyst-15 system in a methanol/toluene mixture under microwave irradiation [171]. Methyl, ethyl, and isopropyl benzoates were prepared from benzaldehyde under irradiation of its alcoholic solutions with a mercury lamp in an oxygen atmosphere; esters were obtained in higher yields in the presence of catalytic amounts of HCl [172]. The formation of primary and secondary alcohol esters was observed after the ozonolysis of a mixture of aldehyde and alcohol in a basic medium [173]. The oxidative esterification of aldehydes was performed using peroxides in the presence of Lewis acids or in the absence of the latter; for example, using oxone in the presence of In(OTf)_3_ [174–175], oxone [174,176], Caro’s acid [177], H_2_O_2aq_ (30%) in the presence of CaCl_2_ or MgCl_2_ [178], H_2_O_2aq_ (50%) [179].

The oxidative coupling of structurally diverse aldehydes (aromatic, α,β-unsaturated, etc.) with hexafluoroisopropanol was carried out in the presence of the oxoammonium salt (4-acetylamino-2,2,6,6-tetramethylpiperidine-1-oxoammonium tetrafluoroborate) and pyridine [180]. The drawback of this method is that it requires a rather complicated and expensive oxidant.

An efficient aerobic cross-dehydrogenative coupling of alcohols and α-carbonyl aldehydes was achieved employing a CuBr/pyridine catalytic system in toluene, only 1.5-fold excess of alcohols over aldehydes was used [181]. The reaction time is 18 h at 90 °C, the yields of α-ketoesters vary from 42 to 88%.

**Scheme 39:**
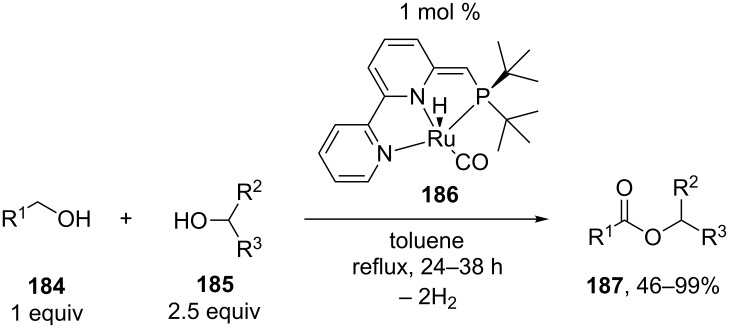
Ruthenium catalyzed cross-dehydrogenative coupling of primary and secondary alcohols.

The unusual C–O cross-coupling of primary alcohols **184** with secondary alcohols **185** in the absence of oxidants was performed in the presence of ruthenium complex **186** as the catalyst; the reaction afforded molecular hydrogen and unsymmetrical ester **187** (Scheme 39) [182].

Cross-coupling products **187** were obtained in high yields; the expected homocoupling of primary alcohols giving symmetrical esters and the dehydrogenation of secondary alcohols yielding ketones were avoided.

### 3 Ketones and 1,3-dicarbonyl compounds as C-reagents in cross-dehydrogenative C–O coupling

Most of cross-dehydrogenative C–O coupling reactions involving the α-position of carbonyl compounds (acetoxylation, alkoxylation, sulfonyloxylation) are based on the use of iodine-containing oxidizing agents. Transition metal salts, such as copper and manganese salts, were less often employed for this purpose.

#### 3.1 Oxidative systems based on iodine compounds

Iodine(III) organic compounds, including those generated in situ from aryl iodides and peracids (for example, *m*-chloroperbenzoic acid (MCPBA) and peracetic acid) are most commonly employed in the oxidative coupling of OH-reagents with carbonyl compounds. Methods were developed for the sulfonyloxylation of ketones, in which iodoarene is generated in situ by the iodination of arene with molecular iodine [183] or NH_4_I [184] in the presence of *m*-chloroperbenzoic acid.

**Scheme 40:**
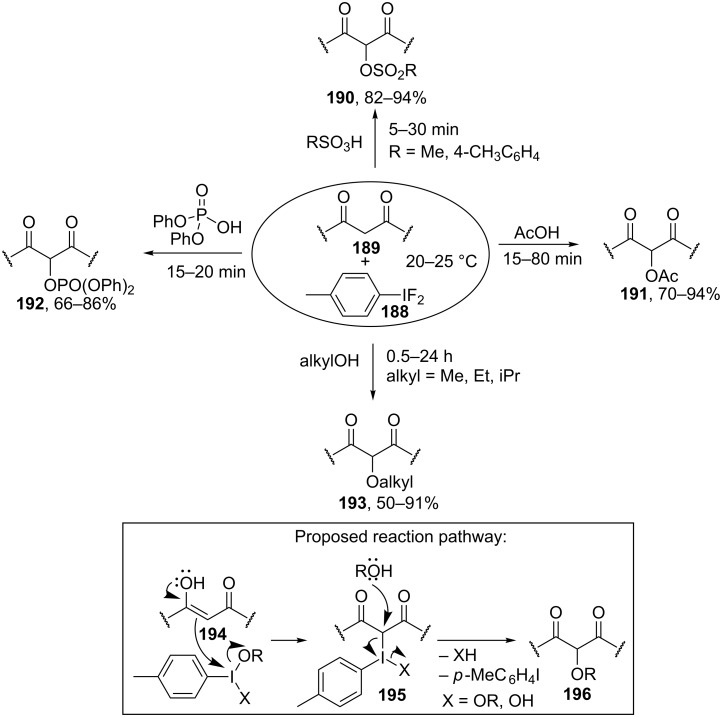
Cross-dehydrogenative C–O coupling reactions of β-dicarbonyl compounds with sulfonic acids, acetic acid, diphenyl phosphate, and alcohols using *p*-(difluoroiodo)toluene.

In the presence of *p*-(difluoroiodo)toluene (**188**), β-dicarbonyl compounds **189** undergo oxidative coupling with various OH-reagents (Scheme 40), such as sulfonic acids (coupling products **190**), acetic acid (products **191**), diphenyl phosphate (products **192**), and alcohols (products **193**) [185]. The reactions with alcohols are most slow. It is suggested that the oxidative C–O coupling at the α-position of carbonyl (or β-dicarbonyl) compounds and various OH-reagents in the presence of iodine(III) compounds proceeds through an ionic mechanism. Thus, the electrophilic iodine atom attacks the enol of carbonyl compound **194** followed by the replacement of the iodine-containing moiety in intermediate **195** by the O-nucleophile to form C–O coupling product **196** (Scheme 40).

Table 6 presents other examples of C–O coupling reactions at the α-position of carbonyl compounds based on the oxidation with iodine(III) compounds or iodoarenes in the presence of peroxides. Chiral iodoarenes, such as **197**, served as the catalysts in the asymmetric C–O coupling of sulfonic acids with ketones. The enantiomeric excess of the product was not higher than 58% due, in particular, to instability of the configuration of the products, α-sulfonyloxy ketones, under the reaction conditions, resulting in the partial racemization [186].

**Table 6:** C–O coupling reactions at the α-position of carbonyl compounds mediated by iodine(III) compounds or iodoarenes in the presence of peroxides.

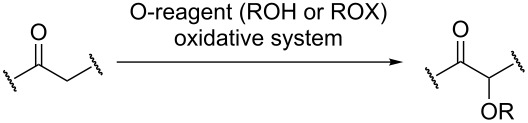				

C-reagent	O-reagent	Oxidative system	Conditions; yields	Ref.

β-diketones, β-keto esters	MeSO_3_H (1 equiv)	PhIO	CHCl_3_, reflux, 2 h; 37–83%	[187]
β-diketones, β-keto esters	MeOH or EtOH (as the solvents)	PhIO, BF_3_·Et_2_O	room temperature, 5 h; 59–67%	[187]
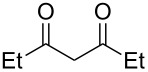	ROH, R = iBu, CMe_2_Et, (CH_2_)_2_CF_3_, (CH_2_)_3_OBn, etc.	PhIO, BF_3_·Et_2_O	CHCl_3_, room temperature, 5 h; yields were not reported	[188]
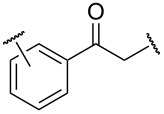	acetoxylation with PhI(OAc)_2_ (1.2 equiv)	PhI(OAc)_2_, Bu_4_NBr, KOH	dioxane, room temperature, 1 h; 74–87%	[189]
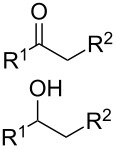 R^1^ = Ar, alkyl R^2^ = H, alkyl, COOMe	RSO_3_H (1.1–5 equiv)	PhI or poly(4-iodostyrene) (cat), MCPBA; KBr_cat_ or TEMPO_cat_ was added to oxidize alcohols	MeCN or CHCl_3_, 50 °C, 5 h; 25–81%	[190]
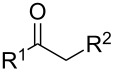 R^1^ = Ar, alkyl, R^2^ = H, alkyl, CO_2_Et	*p*-TsOH (1.1–5 equiv.)	polymer-immobilized iodobenzene, MCPBA (1.1–2.5 equiv), *p*-TsOH	50 °C, 9–16 h; 51–88%	[191]
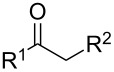 R^1^ = Ar, Et, *t-*Bu, etc. R^2^ = H, alkyl, etc.	acetoxylation with Ac_2_O	PhI, 30% aq H_2_O_2_, Ac_2_O, BF_3_·Et_2_O	30 °C, 7 h; 32–86%	[192]
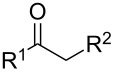 R^1^ = Ar, alkyl, R^2^ = H, alkyl	*p*-TsOH (3–5 equiv)	*p*-MeC_6_H_4_I (1 equiv), oxone (1.5 equiv),	MeCN, 60 °C; 32–100%	[193]
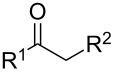 R^1^ = Ar, alkyl, R^2^ = H, alkyl	RSO_3_H (1.5 equiv), R = Ar, alkyl, etc.	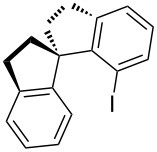 **197** (0.1 equiv) MCPBA (1.5 equiv)	EtOAc, room temperature; 8–41%	[186]

**Scheme 41:**
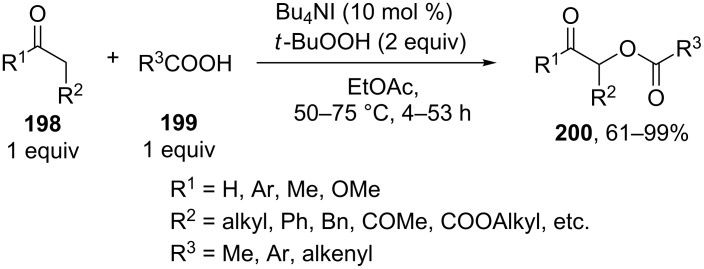
Acyloxylation of ketones, aldehydes and β-dicarbonyl compounds using carboxylic acids and Bu_4_NI/*t-*BuOOH system.

Good results were achieved in the oxidative C–O coupling of ketones, aldehydes and β-dicarbonyl compounds **198** with carboxylic acids **199** in the presence of the Bu_4_NI/*t-*BuOOH system [194] (Scheme 41). The coupling can be accomplished with a wide range of substrates and gives products **200** in high yields, the C-component and the O-component being taken in a ratio of 1:1. The reactions of aldehydes are similar to the reactions of ketones, the aldehyde moiety remaining unchanged. The authors hypothesized that the reaction proceeds through a radical mechanism [194].

The Bu_4_NI/*t-*BuOOH system was employed also in the oxidative coupling of carboxylic acids with β-keto esters [195].

**Scheme 42:**
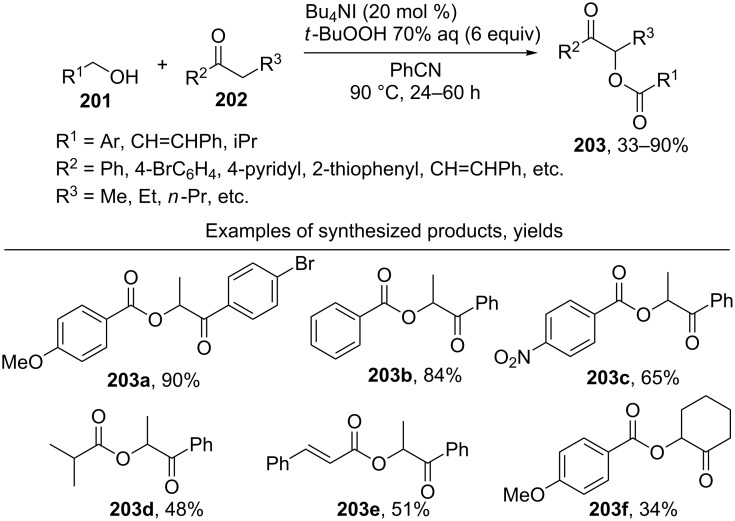
Acyloxylation of ketones using Bu_4_NI/*t-*BuOOH system.

The cross-dehydrogenative C–O coupling of alcohols **201** and ketones **202** in the presence of the Bu_4_NI/*t-*BuOOH system was accomplished to prepare α-acyloxy ketones **203** [196]. Coupling products **203** were synthesized mainly from benzyl alcohols and propiophenone (Scheme 42).

The authors proposed two radical reaction pathways, including the formation of C-radicals from ketones. One of these pathways involves the formation of *tert*-butyl peresters from alcohols. This pathway is confirmed by the fact that, in the presence of Bu_4_NI, *tert*-butyl per(1-naphthylate) gives products **203** in the reaction with propiophenone.

#### 3.2 Oxidative systems based on transition metal compounds

In addition to iodine compounds, transition metal salts, such as copper and manganese salts, were used for the oxidative functionalization at the α-position of carbonyl compounds.

**Scheme 43:**
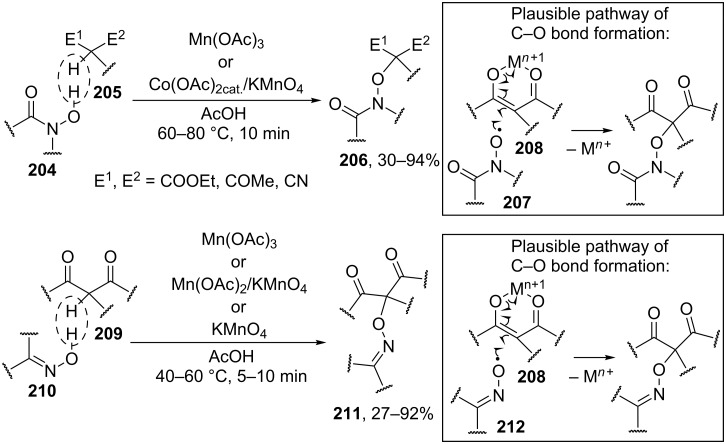
Cross-dehydrogenative C–O coupling of β-dicarbonyl compounds and their heteroanalogues with N-hydroxyimides, *N*-hydroxyamides, and oximes.

*N*-Hydroxyimides and *N*-hydroxyamides **204** are involved in the oxidative C–O coupling with 1,3-dicarbonyl compounds and their hetero analogues, such as 2-substituted malononitriles and cyanoacetic esters, **205** in the presence of oxidants based on manganese, cobalt, and cerium [197]. The best results were achieved with the use of Mn(OAc)_3_ and the Co(OAc)_2(cat)_/KMnO_4_ system (Scheme 43). The yields of products **206** were as high as 94%. It is supposed that the oxidant serves two functions: the generation of *N*-oxyl radicals **207** from *N*-hydroxyimides or *N*-hydroxyamides **204** and the one-electron oxidation of 1,3-dicarbonyl compounds via the formation of complex **208**. Apparently, the oxidative coupling of 1,3-dicarbonyl compounds **209** with oximes **210** occurs via a similar mechanism [198]. The reaction takes place in the presence of various oxidants, such as KMnO_4_, Mn(OAc)_2_/KMnO_4_, Mn(OAc)_3_, MnO_2_, Mn(acac)_3_, Fe(ClO_4_)_3_, Cu(ClO_4_)_2_, Cu(NO_3_)_2_, and (NH_4_)_2_Ce(NO_3_)_6_. Twenty coupling products **211** were prepared in yields of 27–92% using KMnO_4_, Mn(OAc)_3_·2H_2_O, or the Mn(OAc)_2_/KMnO_4_ system. The syntheses do not require excess amounts of reagents. The formation of *N*-oxyl radicals **212** and **207** from oximes and *N*-hydroxyimides was confirmed by ESR spectroscopy [197–198].

**Scheme 44:**
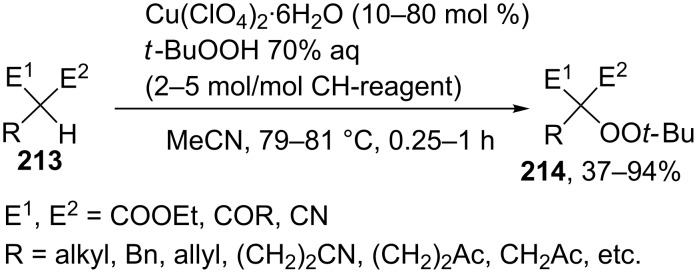
Cross-dehydrogenative C–O coupling of β-dicarbonyl compounds and their heteroanalogues with *t*-BuOOH.

The oxidative coupling of 1,3-dicarbonyl compounds [199] and their hetero analogues [200] **213** with *tert*-butyl hydroperoxides catalyzed by transition metal salts (Cu, Fe, Co, Mn) was achieved. *tert*-Butyl hydroperoxide acts both as the oxidizing agent and the O-component in the coupling. The best results were obtained in the reaction catalyzed by Cu(ClO_4_)_2_·6H_2_O. It was hypothesized that copper serves for the formation of the reactive complex with 1,3-dicarbonyl compounds or their hetero analogues, as well as for the generation of *tert*-butyl peroxide radicals, which react with this complex to give coupling products **214** (Scheme 44).

The related peroxidation reactions with hydroperoxides (*t-*BuOOH, PhMe_2_COOH) in the presence of transition metal salts (cobalt, manganese, or copper) were performed with cyclohexanone, 2-methylcyclohexanone, cyclohexene, 1-octene, cumene, xylene, dimethylaniline, and dioxane [201].

**Scheme 45:**
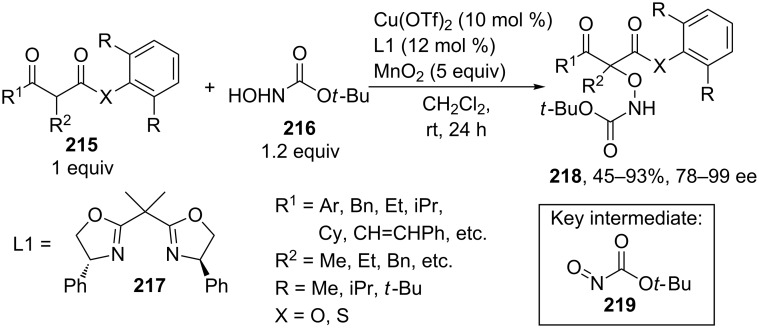
Oxidative C–O coupling of 2,6-dialkylphenyl-β-keto esters and thioesters with *tert*-butyl hydroxycarbamate.

The enantioselective oxidative coupling of 2,6-dialkylphenyl-β-keto esters and thioesters **215** with *tert*-butyl hydroxycarbamate **216** was performed using the Cu(OTf)_2_/chiral ligand **217**/MnO_2_ system (Scheme 45) [202]. Apparently, product **218** is generated via an ionic mechanism involving the generation of electrophilic nitrosocarbonyl intermediate **219** [202].

The improved version of that oxidative coupling method is based on CuCl/Cu(OAc)_2_/ligand/air catalytic system; different β-keto esters and hydroxamic acid derivatives can be used [203].

The acetoxylation at the α’-position of α,β-unsaturated ketones with Mn(OAc)_3_ was studied in detail. It is suggested that manganese(III) acetate causes the generation of C-radicals from ketones followed by the acetoxylation of these radicals. In this reaction, Mn(OAc)_3_ [204–210] or acetic acid [209–210], which was used as the co-solvent, can serve as the source of the acetoxy group. The synthesis is usually performed in benzene. In some cases, the α’-phenylation-α’-acetoxylation was observed due, apparently, to the addition of the C-radical generated from ketone to benzene [204]. It was shown [209] that the α’-acetoxylation of enones occurs with good selectivity in other solvents, such as cyclohexane and acetonitrile, as well.

**Scheme 46:**
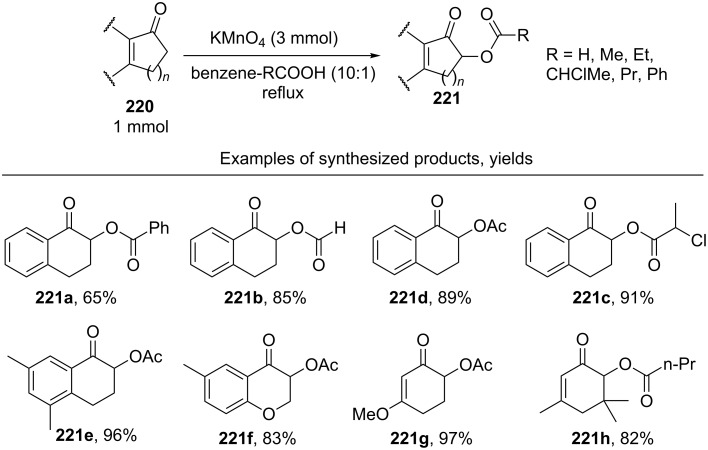
α’-Acyloxylation of α,β-unsaturated ketones using KMnO_4_.

The acyloxylation of enones and aryl ketones **220** with carboxylic acids occurs in benzene in the presence of KMnO_4_ to form coupling products **221** (Scheme 46) [211]. Acids were present in a large excess relative to ketones.

The synthesis was successfully accomplished, in particular, with readily oxidizable formic acid; the corresponding formates (for example, **221b**) were obtained in 61–85% yield [211]. This method was modified by using not carboxylic acids but their mixtures with the corresponding anhydrides and was applied to the α’-acyloxylation of α,β-unsaturated ketones with complex structures with the aim of preparing analogues of natural compounds [212].

### 4 Compounds with an allyl, propargyl, or benzyl group as C-reagents in cross-dehydrogenative C–O coupling

#### 4.1 Palladium- and copper-based oxidative systems

Studies on the acyloxylation at the allylic position of alkenes with palladium(II) complexes started in the 1960s [213]. This type of reactions was considered in more detail in reviews on the palladium complex-catalyzed functionalization of allyl-containing compounds [20–21].

**Scheme 47:**
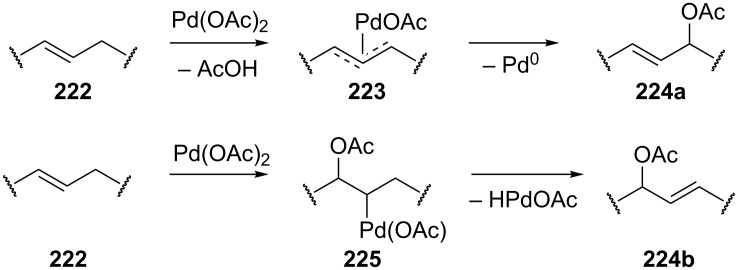
Possible mechanisms of the acetoxylation at the allylic position of alkenes by Pd(OAc)_2_.

It is suggested that the reaction proceeds through the cleavage of the allylic C–H bond in **222** to form π-allyl–palladium complex **223** followed by the nucleophilic attack of acetate to give C–O coupling product **224a** (Scheme 47) [214–217]. The alternative mechanism involves the acetoxypalladation of the double bond in **222** resulting in the formation of intermediate **225** followed by the elimination of HPdOAc to give product **224b** [213]. The first mechanism of the acetoxylation was confirmed by the data on the acetoxylation of 1,2-dideuteriocyclohexene [215], as well as by the detection of the π-allyl–palladium intermediate [217]. However, it cannot be ruled out that the acetoxylation can occur via the second mechanism under certain conditions (Scheme 47) [21,213].

**Scheme 48:**
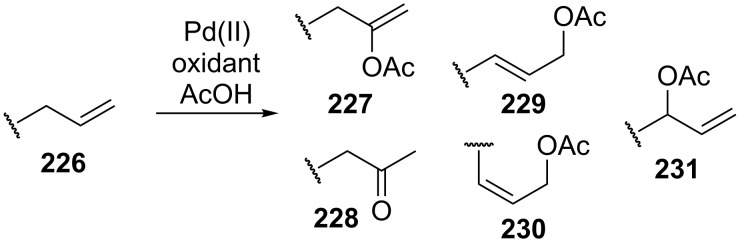
Products of the oxidation of terminal alkenes by Pd(II)/AcOH/oxidant system.

The palladium-complex-catalyzed acetoxylation of terminal alkenes **226** under non-optimized conditions can afford a large number of products: vinyl acetate **227** and methyl ketone **228** (Wacker reaction), *E* and *Z* isomers of linear allyl ethers **229** and **230**, and branched allyl ether **231** (Scheme 48) [218].

It was found that the selectivity of the reaction can be controlled by changing the polarity of the solvent. The reactions of terminal alkens with the Pd(OAc)_2_/benzoquinone system in a mixture of DMSO and AcOH as the solvent selectively produced linear *E*-allyl acetates in 50–65% yield [218]. The reaction in acetic acid affords methyl ketone and vinyl acetate; branched allyl ether is formed as the major product in the CH_2_Cl_2_/AcOH system using the 1,2-bis (benzylsulfinyl)ethane ligand [218].

**Scheme 49:**
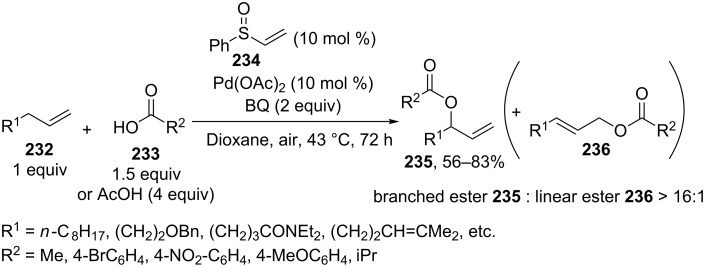
Acyloxylation of terminal alkenes with carboxylic acids.

The acyloxylation of terminal alkenes **232** with carboxylic acids **233** in the presence of 1,4-benzoquinone (BQ) as the oxidant, vinyl phenyl sulfoxide ligand **234**, and Pd(OAc)_2_ resulted in the selective formation of branched allyl esters **235** [217]. This reaction afforded linear esters **236** as byproducts. Apparently, ligand **234** serves for the formation of the π-allyl–palladium intermediate, and benzoquinone mediates the subsequent reductive elimination to give product **235** (Scheme 49) [217].

The Pd(OAc)_2_·[1,2-bis(phenylsulfinyl)ethane]-catalyzed enantioselective acetoxylation of terminal alkenes was accomplished in the presence of a chiral Lewis acid; ee = 45–63%; the reaction was performed with a small excess of AcOH (1.1 equiv) in ethyl acetate using benzoquinone as the oxidant [219].

The Pd(OAc)_2_-catalyzed acetoxylation of terminal alkenes employing 4,5-diazafluorenone as the ligand can be performed in the presence of oxygen as the oxidizing agent (1 atm). The reaction gave linear allyl esters. The authors reported that this ligand facilitates the reductive elimination of the allyl ester from the π-allyl–palladium intermediate [220]. Linear *E*-allyl acetates were synthesized also by the acetoxylation of terminal alkenes with the PdCl_2_/NaOAc/AcOH/O_2_ system (5 atm) in *N*,*N*-dimethylacetamide [221]. The Wacker reaction giving methyl ketones occurs when using water instead of sodium acetate and acetic acid, all other conditions being the same [221].

**Scheme 50:**
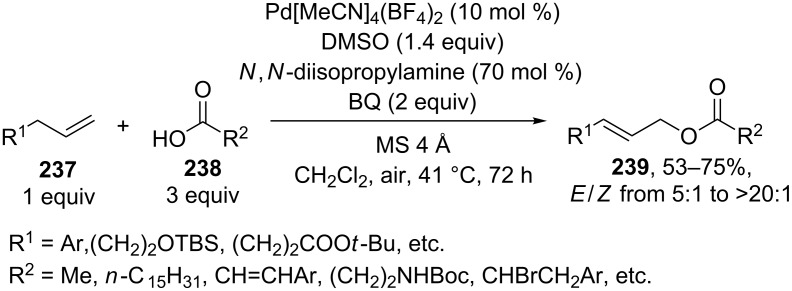
Synthesis of linear *E-*allyl esters by cross-dehydrogenative coupling of terminal alkenes wih carboxylic acids.

The coupling of terminal alkenes **237** with carboxylic acids having complex structures **238** resulted in the selective formation of linear *E-*allyl esters **239** (Scheme 50) [222]. These reactions afford a *Z* isomer of linear ester and branched allyl ester as byproducts.

Similar results were obtained in the coupling of terminal alkenes in the presence of lithium hydroxide as the base in carboxylic acid acting as the OH-reagent or in a carboxylic acid/acetonitrile mixture; the coupling was performed with acetic, propionic, isobutyric, and pivalic acids [223].

**Scheme 51:**
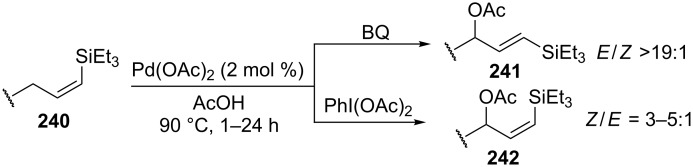
Pd(OAc)_2_-catalyzed acetoxylation of *Z-*vinyl(triethylsilanes).

It was shown that the stereoselectivity of the Pd(OAc)_2_-catalyzed acetoxylation of *Z-*vinyl(triethylsilanes) **240** can be controlled using benzoquinone or (diacetoxyiodo)benzene as the oxidant [224]. The reaction with benzoquinone affords the *E* isomer of C–O coupling product **241**, whereas *Z* isomer **242** is formed as the major product in the case of (diacetoxyiodo)benzene (Scheme 51).

**Scheme 52:**
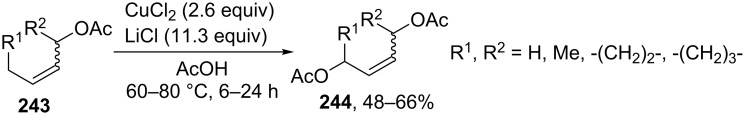
α’-Acetoxylation of α-acetoxyalkenes with copper(II) chloride in acetic acid.

The α’-acetoxylation of α-acetoxyalkenes **243** with copper(II) chloride in acetic acid was reported [225] (Scheme 52). Alkenes free of the α-acetoxy group undergo *trans*-chlorination of the double bond in the presence of CuCl_2_. α,α’-Diacetoxyalkenes **244** were synthesized from alkenes with the additional use of the PdCl_2_/NaOAc/DDQ or Pd(OAc)_2_/benzoquinone/MnO_2_ reagents.

**Scheme 53:**
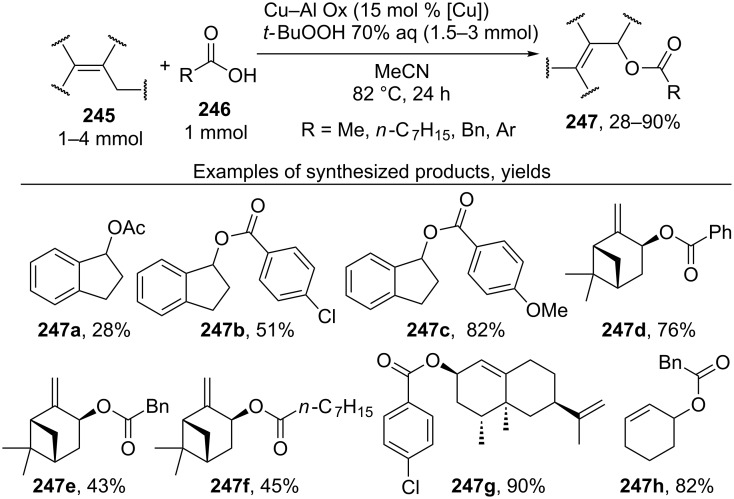
Oxidative acyloxylation at the allylic position of alkenes and at the benzylic position of alkylarenes with carboxylic acids.

The oxidative acyloxylation at the allylic position of alkenes and at the benzylic position of some alkylarenes **245** with carboxylic acids **246** was accomplished in the presence of *tert*-butyl hydroperoxide and mixed copper aluminum oxide as the heterogeneous catalyst (Scheme 53) [226]. The catalyst was prepared from CuCl_2_ and AlCl_3_·6H_2_O by the co-precipitation from an aqueous solution in the presence of NaOH and Na_2_CO_3_. Allyl esters **247** with various structures were synthesized. The reactions were carried out taking alkene and carboxylic acid in a ratio of 1:1 or in the presence of an excess of alkene.

**Scheme 54:**
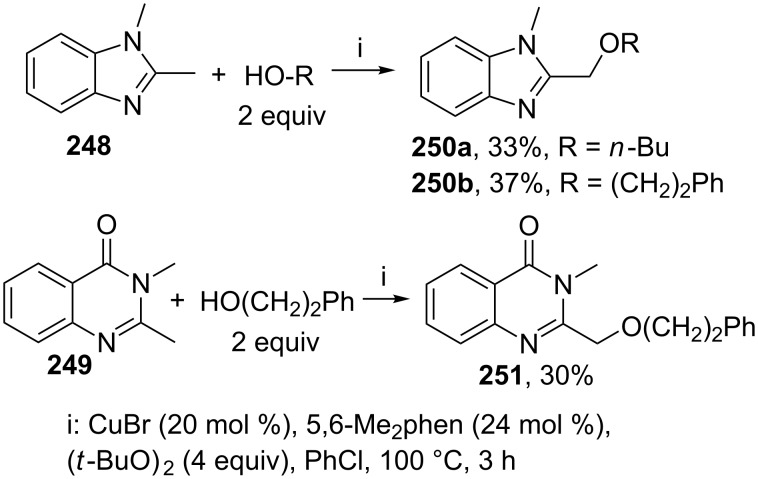
Copper-catalyzed alkoxylation of methylheterocyclic compounds using di-*tert*-butylperoxide as oxidant.

The intermolecular alkoxylation of methylheterocyclic compounds **248**, **249** using the CuBr/5,6-dimethyl-1,10-phenanthroline (5,6-Me_2_phen)/(*t*-BuO)_2_ oxidative system was shown by a few examples [227]. The yields of coupling products **250**, **251** were not higher than 37% (Scheme 54).

**Scheme 55:**
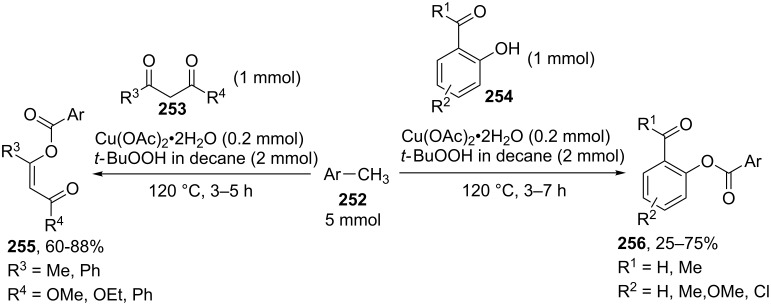
Oxidative C–O coupling of methylarenes with β-dicarbonyl compounds or phenols.

Methylarenes **252** were introduced into the oxidative C–O coupling with β-dicarbonyl compounds **253** and phenols **254** [228]. The coupling afforded structures **255** and **256**. The method is applicable to phenols containing an aldehyde group, which was not oxidized in the course of the reaction (Scheme 55).

**Scheme 56:**
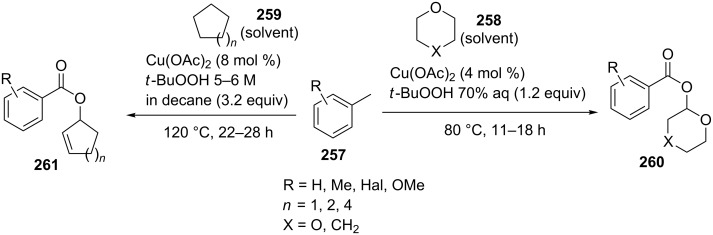
Copper-catalyzed esterification of methylbenzenes with cyclic ethers and cycloalkanes.

Oxidative C–O coupling of methylarenes **257** with cyclic ethers **258** and cycloalkanes **259** afforded α-acyloxy ethers **260** and allyl esters **261**, respectively. The reactions were accomplished using the Cu(OAc)_2_/*t-*BuOOH oxidative system at low conversions of methylarenes in the corresponding ether or cycloalkane as solvent [229] (Scheme 56).

The proposed mechanism includes the copper-mediated generation of *tert*-butyl peresters from *t*-BuOOH and methylarenes and the formation of final products via intermolecular coupling of O-centered and C-centered radicals. Related oxidative C–O coupling reactions of alkanes with aromatic aldehydes [169] and carboxylic acids are discussed in sections 2.5 and 6, respectively.

The 4-methyl group of 2,4,6-trimethylphenol was selectively methoxylated in methanol in the presence of a copper(II) complex and hydrogen peroxide [230]; the similar alkoxylation reaction was performed with a stoichiometric amount of copper(II) chloride [231].

**Scheme 57:**
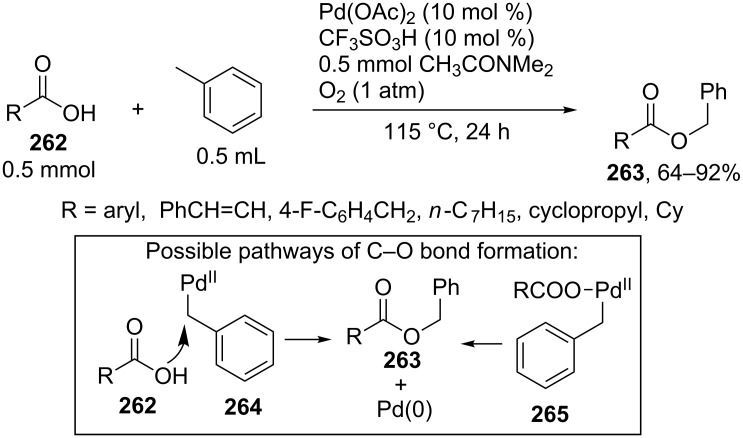
Oxidative C–O coupling of carboxylic acids with toluene catalyzed by Pd(OAc)_2_.

The benzylation of aryl-, alkyl-, and cycloalkylcarboxylic acids **262** in toluene was accomplished in the presence of the Pd(OAc)_2_/CF_3_SO_3_H/dimethylacetamide/O_2_ system (Scheme 57) [232]. Apparently, the reaction proceeds through an ionic mechanism involving the cleavage of the C–H bond of toluene with Pd(II). The product is formed either via the nucleophilic attack of carboxylic acid on Pd(II) complex **264** or as a result of the reductive elimination of product **263** from complex **265**. It is supposed that dimethylacetamide promotes the reoxidation of Pd(0) to Pd(II) with oxygen and suppresses the aggregation of Pd(0), while trifluoromethanesulfonic acid facilitates the cleavage of the C–H bond of toluene through the formation of cationic Pd(II) compounds [232].

The gas-phase aerobic acetoxylation of toluene with acetic acid catalyzed by TiO_2_-, γ-Al_2_O_3_-, SiO_2_-, and ZrO_2_-supported Pd–Sb particles was also reported [233–236].

#### 4.2 Reactions catalyzed by tetrabutylammonium iodide

**Scheme 58:**
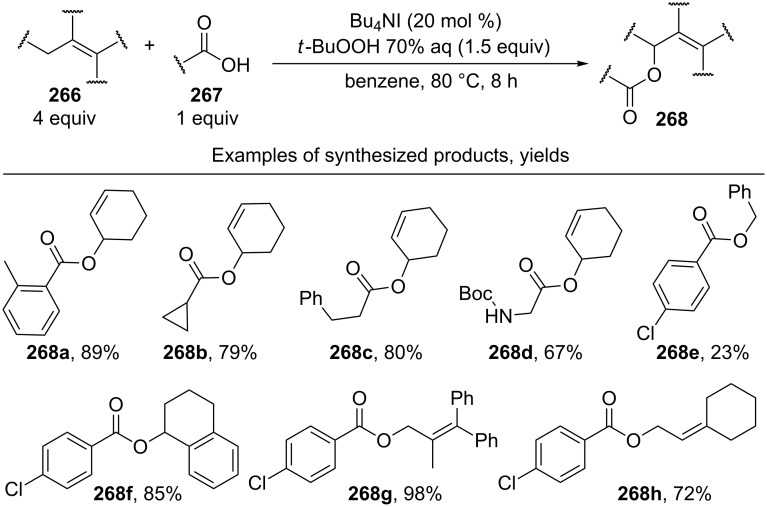
Oxidative acyloxylation at the allylic position of alkenes with carboxylic acids using the Bu_4_NI/*t-*BuOOH system.

The oxidative acyloxylation at the allylic position of alkenes **266** with carboxylic acids **267** was performed using the Bu_4_NI/*t-*BuOOH system to prepare esters **268** [237]. Apparently, the reaction proceeds through a radical mechanism, involving the hydrogen atom abstraction from the allylic position of alkene **266** with the *tert*-butyl peroxyl or *tert*-butoxyl radical; the authors suggested that the new C–O bond is formed as a result of the cross-recombination of the allyl and carboxyl radicals (Scheme 58) [237].

**Scheme 59:**
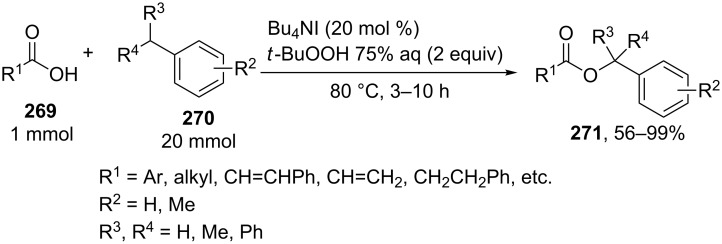
Cross-dehydrogenative C–O coupling of carboxylic acids with alkylarenes using the Bu_4_NI/*t-*BuOOH system.

The coupling of carboxylic acids **269** with alkylarenes **270** occurs analogously to the coupling with alkenes (Scheme 59) [141,237]. It is supposed that [Bu_4_N]^+^IO^−^ or [Bu_4_N]^+^[IO_2_]^−^ generated in the Bu_4_NI/*t-*BuOOH system act as active oxidizing agents; the fact that the reaction proceeds via the hydrogen atom abstraction from the benzylic position to form the benzyl radical is confirmed by a number of experiments, including the trapping of the benzyl radical by the TEMPO radical [141]. The authors hypothesized that the benzyl radical is oxidized to the benzyl cation, which undergoes nucleophilic attack by the carboxylate anion to give cross-dehydrogenative C–O coupling product **271**. It was shown that 1-iodo-1-phenylethane does not react with carboxylic acid under these reaction conditions [141].

The oxidative C–O coupling of alkylarenes with aromatic benzyl alcohols [140] and aldehydes [142] in the presence of the Bu_4_NI/*t-*BuOOH system is considered in section 2.4 (Scheme 27 and Scheme 28).

**Scheme 60:**
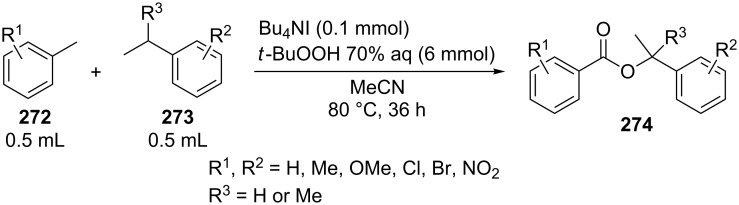
Oxidative C–O cross-coupling of methylarenes with ethyl or isopropylarenes.

The synthesis of symmetrical esters from methylarenes and of unsymmetrical esters **274** from methylarenes **272** and ethylarenes **273** in the presence of the Bu_4_NI/*t-*BuOOH system was documented (Scheme 60). The reaction was accomplished at low conversions of alkylarenes [238]. It was suggested that methylarene is oxidized to carboxylic acid and reacts with the benzylic carbocation generated from the second methylarene molecule or from ethylarene through the hydrogen atom abstraction followed by the oxidation.

The achievement of the cited study is that the authors succeeded in performing the selective oxidative C–O cross-coupling of methylarenes **272** with ethylarenes **273** and excluded side processes, in which symmetrical esters could be generated from methylarenes **272** (the formation of only small amounts of symmetrical esters was observed; 2–5% yield), as well as the oxidation of ethylarene **273** to aryl methyl ketone.

**Scheme 61:**
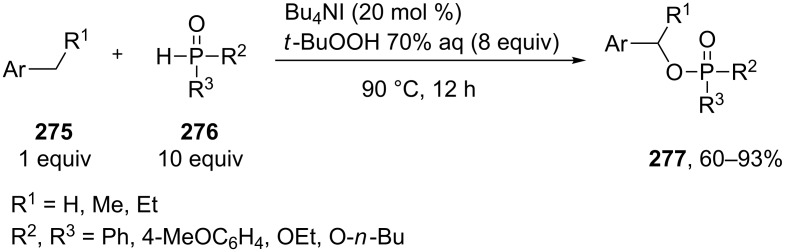
Phosphorylation of benzyl C–H bonds using the Bu_4_NI/*t*-BuOOH oxidative system.

Phosphorylation of benzyl C–H bonds of alkylarenes **275** using the Bu_4_NI/*t*-BuOOH oxidative system was documented to afford products **277** (Scheme 61) [239]. In this process two bonds (C–H and P–H) are cleaved and two new bonds (C–O and P–O) are formed. The proposed mechanism includes benzyl radical formation, oxidation of the latter to the benzylic cation, and nucleophilic attack of phosphate which is formed by the oxidation of the P–H bond in **276**.

**Scheme 62:**
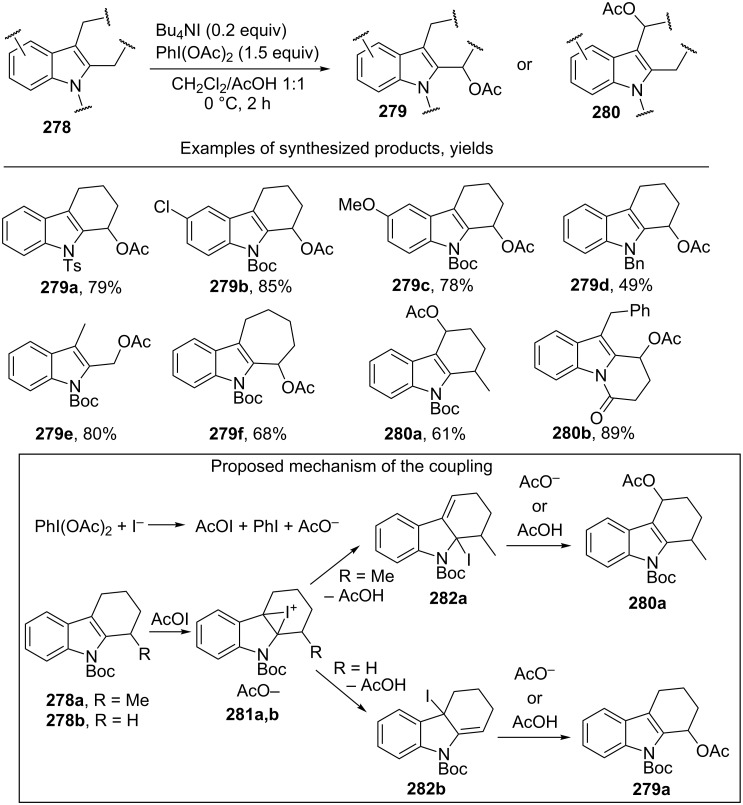
Selective C–H acetoxylation of 2,3-disubstituted indoles.

2,3-Disubstituted indoles **278** are acetoxylated in the presence of the Bu_4_NI/PhI(OAc)_2_ system in a CH_2_Cl_2_/AcOH solvent mixture. In most cases, the reaction resulted in the regioselective introduction of the acetoxy group at one of the two possible positions resulting in the formation of product **279** or **280** (Scheme 62) [240].

The authors supposed that indoles undergo iodination (as exemplified by indoles **278a**,**b**) with AcOI, which is generated from PhI(OAc)_2_ and the iodide anion, through the formation of intermediates **281a**,**b** to give one of the two allyl iodides (**282a** or **282b**); the nucleophilic attack of acetic acid or the acetate anion (S_N_’ nucleophilic substitution) affords products **280a** or **279a**.

#### 4.3 Reactions using 2,3-dichloro-5,6-dicyano-1,4-benzoquinone (DDQ)

The common feature of the cross-dehydrogenative C–O coupling reactions that take place in the presence of DDQ is that C-reagents should contain conjugated systems without strong electron-withdrawing groups.

**Scheme 63:**
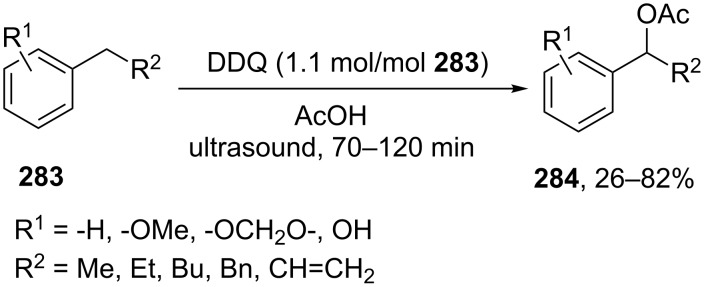
Acetoxylation of benzylic position of alkylarenes using DDQ as oxidant.

For example, the acetoxylation of alkylarenes **283** with DDQ in acetic acid under ultrasound irradiation produced acetates **284** (Scheme 63) [241].

The ultrasound irradiation results in a considerable increase in the selectivity of the reaction. The proposed mechanism involves the generation of acetoxyl radicals and DDQ-H radicals in the reaction of DDQ with acetic acid, and the latter radicals abstract a hydrogen atom from the benzylic position of alkylarene. The target product is formed as a result of the recombination of benzyl and acetoxyl radicals. The reactions with methylarenes were not performed; alkylarenes containing electron-withdrawing substituents (Br, NO_2_) were not involved in the oxidative coupling.

**Scheme 64:**
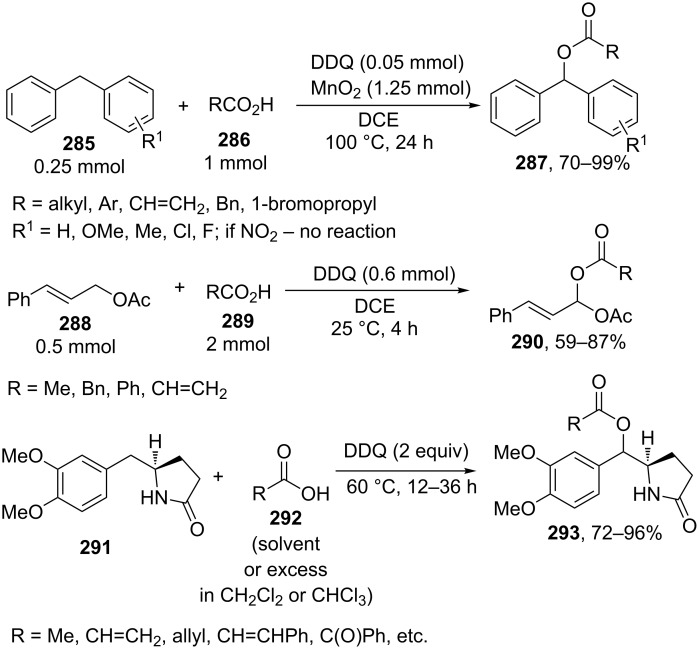
C–H acyloxylation of diarylmethanes, 3-phenyl-2-propen-1-yl acetate and dimethoxyarene using DDQ.

The oxidative coupling of diarylmethanes **285** with carboxylic acids **286** takes place in the presence of the DDQ_cat_/MnO_2_ system (Scheme 64) [242]. It is suggested that DDQ oxidizes diarylmethanes to diarylmethyl cations, which react with carboxylic acids to form esters **287**. Manganese dioxide serves for the oxidation of the reduced form of the catalyst DDQH_2_ to DDQ. The drawback of this method is that the reaction center of the C-component should contain two aryl groups and that a fourfold excess of carboxylic acids (O-components) is required. In the cited study, it was also reported that the coupling of carboxylic acids **289** with 3-phenyl-2-propen-1-yl acetate (**288**) in the presence of DDQ gives allyl esters **290**. DDQ was used also for the acyloxylation at the benzylic position of dimethoxyarene **291** with carboxylic acids **292** to prepare esters **293** (Scheme 64) [243].

The benzylic position of β-phenylethylbenzamides was acetoxylated with DDQ in acetic acid at 80 °C; the reaction readily proceeds if the phenyl ring contains activating methoxy groups; in the case of unsubstituted β-phenylethylbenzamide, the conversion was incomplete [244]. (*p*-Isopropoxyphenyl)acetic acid esters were stereoselectively acetoxylated with DDQ despite the presence of an electron-withdrawing ester group in the vicinity of the reaction center [245].

**Scheme 65:**
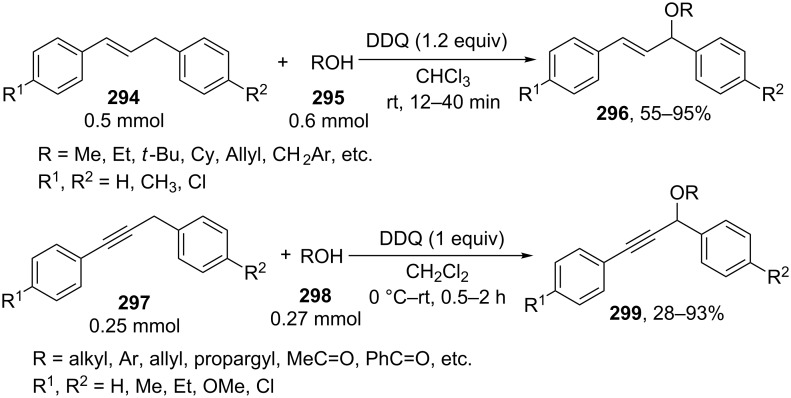
Cross-dehydrogenative C–O coupling of 1,3-diarylpropylenes and 1,3-diarylpropynes with alcohols.

The oxidative C–O coupling of 1,3-diarylpropylenes **294** with alcohols **295** was accomplished in the presence of DDQ [246]; the reaction was completed at room temperature in a period of time shorter than one hour; a mixture of two isomeric coupling products **296** was obtained in the case of different substituents R^1^ and R^2^. Under similar conditions, the oxidative coupling of 1,3-diarylpropynes **297** with alcohols, phenols, and carboxylic acids **298** afforded coupling products **299** (Scheme 65) [247].

**Scheme 66:**
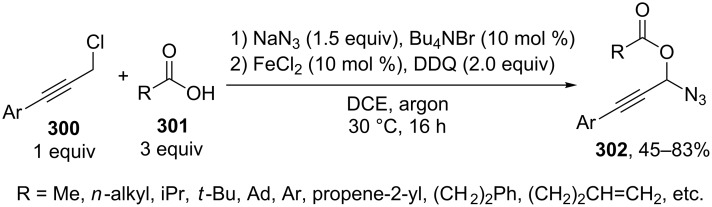
One-pot azidation and C–H acyloxylation of 3-chloro-1-arylpropynes.

A method was developed for the one-pot synthesis of 3-acyloxy-3-azido-1-aryl-1-propynes **302** from 3-chloro-1-arylpropynes **300** involving the acyloxylation of the methylene group with carboxylic acids **301** under the action of the FeCl_2_/DDQ system (Scheme 66) [248]. In the absence of iron salts, the reaction is less efficient.

**Scheme 67:**
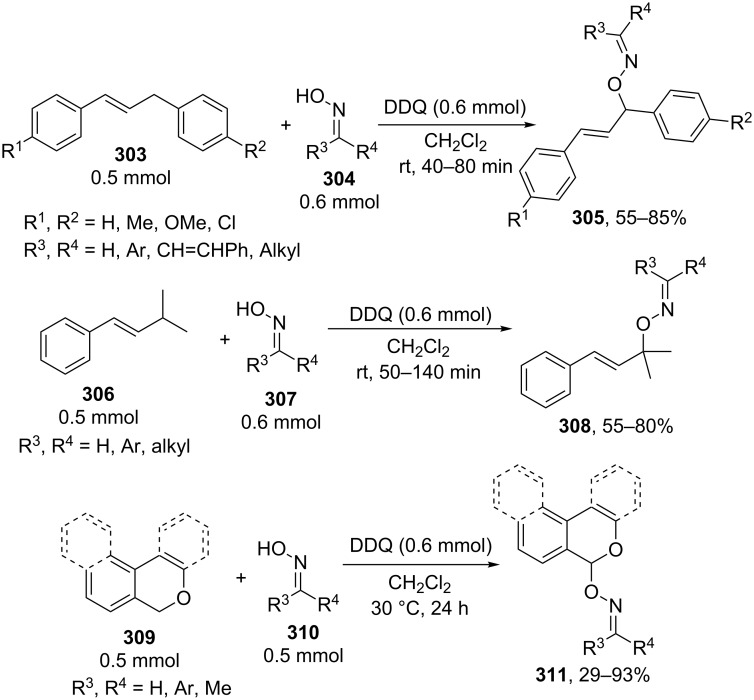
Cross-dehydrogenative C–O coupling of 1,3-diarylpropylenes, (*E*)*-*1-phenyl-2-isopropylethylene and isochromanes with oximes.

The cross-dehydrogenative C–O coupling reactions with the use of oximes as O-reagents in the presence of DDQ were documented. 1,3-Diarylpropylenes **303**, as well as (*E*)*-*1-phenyl-2-isopropylethylene (**306**) are involved in the oxidative coupling with oximes **304** and **307** in the presence of DDQ [249]. Under similar conditions, oximes **310** are involved in the coupling with isochromanes **309** [250] (Scheme 67).

It was suggested that carbocations are generated from C-reagents under the action of DDQ, and these carbocations undergo nucleophilic attack by oxime anions to form coupling products **305**, **308**, and **311** [249–250]. *N*-Hydroxyphthalimide was also employed as the O-reagent, the yield of the coupling product with isochromane was 62% [250]. DDQ was also used in the acetoxylation at the benzylic position of *p*-alkoxyalkylarenes and *p*-alkylphenols in acetic acid [251].

#### 4.4 Reactions with *N*-hydroxyphthalimide

**Scheme 68:**
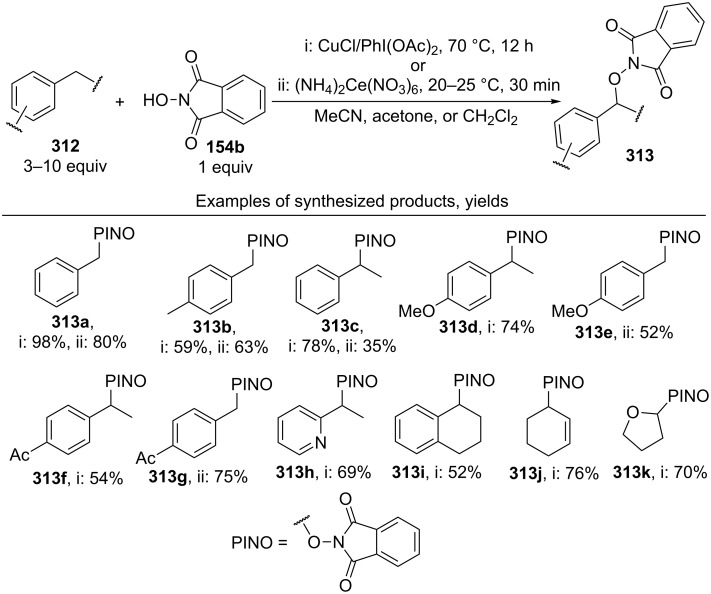
Cross-dehydrogenative C–O coupling of alkylarenes and related compounds with *N*-hydroxyphthalimide.

The oxidative C–O coupling of alkylarenes and related compounds **312** with *N*-hydroxyphthalimide (**154b**) was performed using CuCl/PhI(OAc)_2_ [252] or (NH_4_)_2_Ce(NO_3_)_6_ [253] as the oxidizing agent (Scheme 68). The reaction in the presence of the CuCl/PhI(OAc)_2_ system gives somewhat higher yields of coupling products **313** compared with (NH_4_)_2_Ce(NO_3_)_6_, but it requires higher temperature, a longer reaction time, and relatively larger excesses of alkylarene. Apparently, the C–O bond is formed as a result of the recombination of benzyl and phthalimide-*N*-oxyl radicals. Alkylarenes should be taken in an excess amount to achieve high yields. The coupling of *N*-hydroxyphthalimide with ethers and alkenes proceeds in a similar fashion [252].

It was reported that such oxidative coupling reactions with NHPI and alkylarenes can proceed through the generation of phthalimide-*N*-oxyl radicals from NHPI in the presence of Pb(OAc)_4_; however, the yields of the products were not published [254]. Later on, it was shown that cross-dehydrogenative coupling of NHPI with toluene proceeds in the presence of a variety of oxidants, (NH_4_)_2_Ce(NO_3_)_6_, PhI(OAc)_2_, KMnO_4_, Mn(OAc)_3_·2H_2_O, Pb(OAc)_4_, or Co(OAc)_2_/O_2_ to afford *N*-benzyloxyphthalimide in yields of 11–75% [255]. The oxidative coupling of NHPI with cyclohexene and cyclooctene using NaIO_4_ in the CH_2_Cl_2_/H_2_O mixture in the presence of silica gel was documented [256]; the coupling products were only partially characterized (the NMR spectra of the crude compounds were reported). In another study, the oxidative coupling of NHPI with cyclic and acyclic alkenes was performed in the presence of (NH_4_)_2_Ce(NO_3_)_6_, Pb(OAc)_4_, or anthraquinone; in the case of metal-containing oxidizing agents, the radical reaction mechanism is suggested along with the competitive ionic mechanism; the reaction products and procedures for their synthesis were not completely characterized [257].

**Scheme 69:**
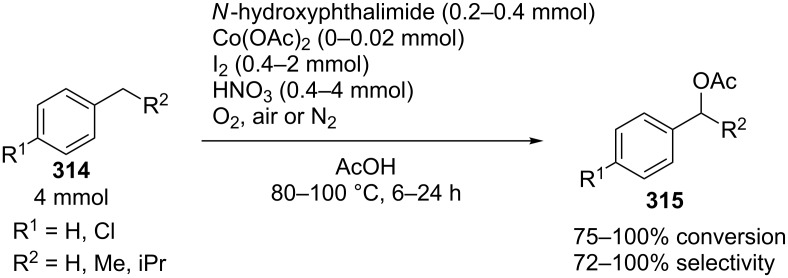
Acetoxylation at the benzylic position of alkylarenes mediated by *N*-hydroxyphthalimide.

The acetoxylation at the benzylic position of alkylarenes **314** in acetic acid was performed using the *N*-hydroxyphthalimide/iodine/nitric acid system; in some experiments, Co(OAc)_2_ was employed as the cocatalyst (Scheme 69) [258]. Oxygen or nitric acid acts as the oxidant.

In the presence of oxidizing agents, phthalimide-*N*-oxyl radicals are generated from NHPI, and these radicals abstract a hydrogen atom from the benzylic position of alkylarene **314**; the resulting benzyl radical is trapped by iodine to form iodide followed by the replacement of iodine by acetic acid to give product **315**. The proposed mechanism is confirmed by the fact that the reactions of alkylarenes containing electron-withdrawing substituents in the benzene ring afford benzyl iodides rather than the corresponding acetates.

#### 4.5 Other reactions

**Scheme 70:**
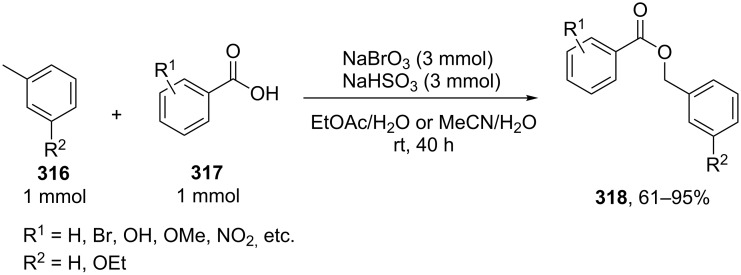
C–O coupling of methylarenes with aromatic carboxylic acids employing the NaBrO_3_/NaHSO_3_ system.

The C–O coupling of methylarenes **316** with aromatic carboxylic acids **317** in the presence of the NaBrO_3_/NaHSO_3_ system was described [259]. The synthesis was performed at room temperature with aromatic carboxylic acids **317** containing both electron-donating and -withdrawing groups; methylarene **316** (toluene or 3-ethoxytoluene), and carboxylic acid **317** were taken in a ratio of 1:1. It was suggested that benzyl bromides are formed in situ followed by the generation of products **318** through the nucleophilic attack by carboxylic acid (Scheme 70).

**Scheme 71:**
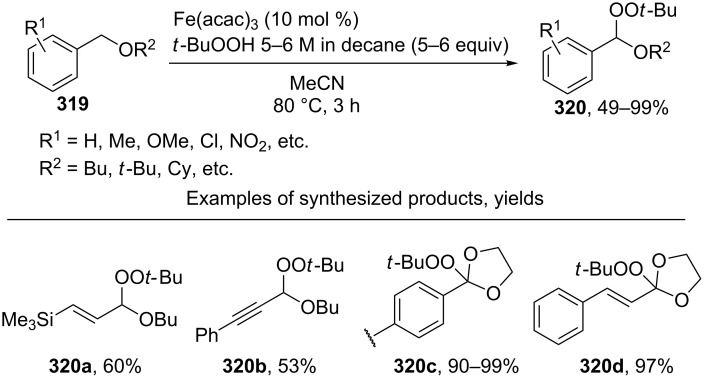
*tert*-Butyl peroxidation of allyl, propargyl and benzyl ethers catalyzed by Fe(acac)_3_.

The *tert*-butyl peroxide group was introduced into allyl, propargyl and benzyl ethers **319** in the presence of the Fe(acac)_3_/*t-*BuOOH system [260]. It was suggested that a hydrogen atom is abstracted from the CH-reagent by *tert*-butylperoxyl and *tert*-butoxyl radicals generated from *tert*-butyl hydroperoxide in the presence of Fe(acac)_3_; the resulting C-radical is oxidized with Fe(III) to the carbocation, and the latter is subjected to nucleophilic attack by *tert*-butyl hydroperoxide to give product **320** (Scheme 71).

The *tert*-butyl peroxidation at the allylic and benzylic positions of cyclohexene and ethylbenzene with *tert*-butyl hydroperoxide catalyzed by nanosized CeO_2_ proceeds with low yields [261].

The acetoxylation of alkylarenes in acetic acid was performed using the CeO_2_/NaBrO_3_ [262], LiBr/NaIO_4_ [263], and H_5_PV_2_Mo_10_O_40_/NaNO_3_ systems [264]. The methyl group of 1,2,3-trimethoxy-5-methylbenzene in acetic acid was acetoxylated with 65% nitric acid [265]. *N*-Benzylphthalimides were acetoxylated at the benzylic position with the *N*-bromosuccinimide/NaOAc/HOAc system by heating under reflux in chlorobenzene for 12 h [266].

The alkoxylation [267–268] or acyloxylation [267,269] at the allylic position of alkenes occurs in the presence of selenium-based oxidizing agents, such as ArSeSeAr_cat._/S_2_O_8_^2−^ [267–268] or SeO_2_ [269]. It is suggested that the reactions proceed through electrophilic attack of selenium on the C=C double bond [268–269].

### 5 Ethers, amines, and amides as C-reagents in cross-dehydrogenative C–O coupling

#### 5.1 Reactions catalyzed by tetrabutylammonium iodide

**Scheme 72:**
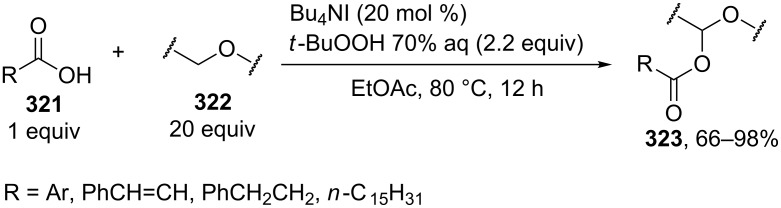
Cross-dehydrogenative C–O coupling of ethers with carboxylic acids mediated by Bu_4_NI/*t-*BuOOH system.

The Bu_4_NI/*t-*BuOOH system proved to be efficient in the oxidative C–O coupling of ethers **322** with carboxylic acids **321** (Scheme 72) [270]. The coupling was performed with ethers taken in a 20-fold excess relative to carboxylic acids. It is supposed that the *tert*-butoxyl radical, which is generated in the Bu_4_NI/*t-*BuOOH system, abstracts a hydrogen atom from the α-position of the ether, and the resulting C-radical is oxidized to the carbocation, which reacts with the carboxylate anion to form coupling product **323**.

**Scheme 73:**
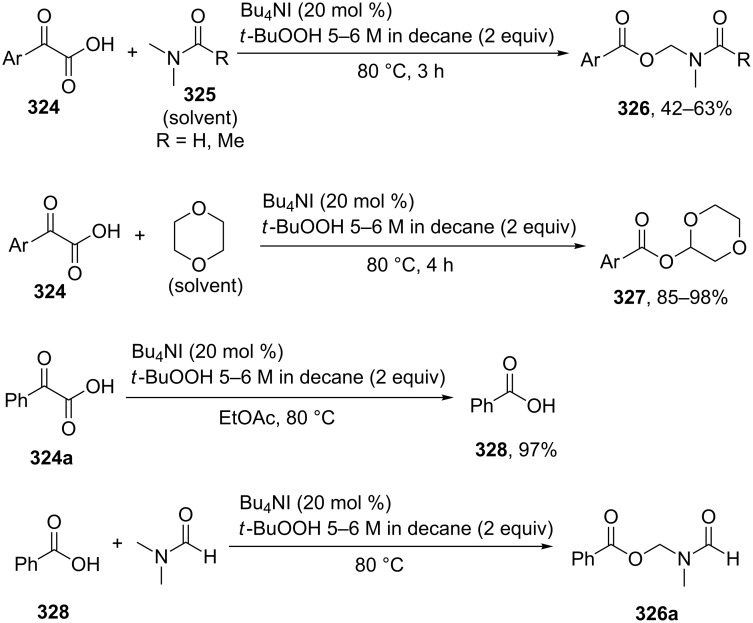
Oxidative acyloxylation of dimethylamides and dioxane with 2-aryl-2-oxoacetic acids accompanied by the decarboxylation.

The Bu_4_NI/*t-*BuOOH system was applied to perform the oxidative acyloxylation of dimethylamides **325** and dioxane with 2-aryl-2-oxoacetic acids **324** accompanied by the decarboxylation [271]. It was shown that, in the presence of the Bu_4_NI/*t-*BuOOH system, 2-oxo-2-phenylacetic acid (**324a**) is transformed into benzoic acid (**328**) followed by the coupling of the latter with dimethylformamide to give product **326a**. Hence, it is supposed that arylcarboxylic acids are intermediates in the synthesis of compounds **326** and **327** (Scheme 73).

**Scheme 74:**
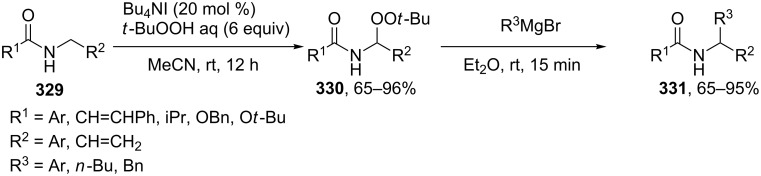
*tert*-Butyl peroxidation of *N*-benzylamides and *N*-allylbenzamide using the Bu_4_NI/*t-*BuOOH system.

The peroxidation of *N*-benzylamides and *N*-allylbenzamide **329** with the Bu_4_NI/*t-*BuOOH system (Scheme 74) was documented [272]. The *tert*-butyl peroxide group in products **330** can be replaced with an aryl, benzyl, or alkyl group by the reaction with the corresponding Grignard reagent to afford products **331**.

#### 5.2 Reactions catalyzed by transition metal salts

**Scheme 75:**
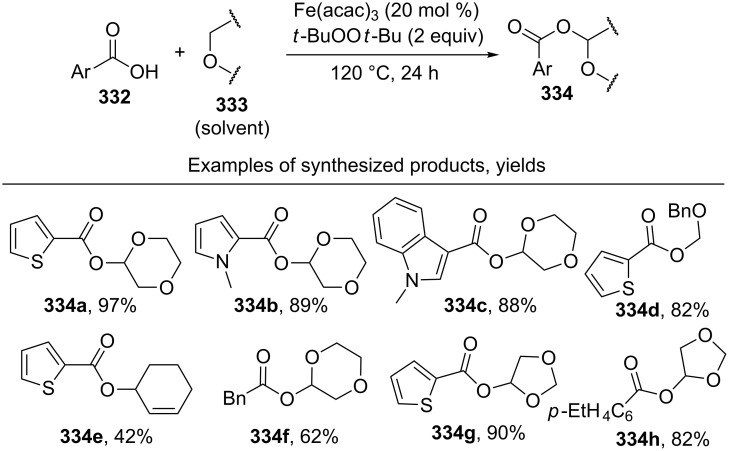
Cross-dehydrogenative C–O coupling of aromatic carboxylic acids with ethers using Fe(acac)_3_ as catalyst and di-*tert*-butyl peroxide as oxidant.

The oxidative coupling of aromatic carboxylic acids **332** with ethers **333**, which served as the solvents, was accomplished using the Fe(acac)_3_/di-*tert*-butyl peroxide system to prepare α-acyloxy ethers **334** (Scheme 75) [273].

In addition to aromatic carboxylic acids **332**, the coupling was performed with phenylacetic acid. The reaction takes place both with ethers and cyclohexene. In almost all syntheses, one of the following two C-reagents was used: 1,4-dioxane or 1,3-dioxolane. It is interesting that the oxidative coupling of carboxylic acids with 1,3-dioxolane occurs at the 4-poisition of dioxolane (with one adjacent oxygen atom) rather than at the 2-position (with two adjacent oxygen atoms), which is usually considered to be more activated for the hydrogen atom abstraction.

**Scheme 76:**
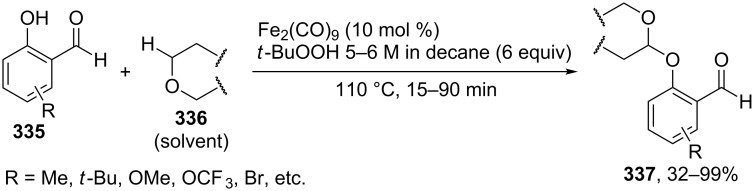
Cross-dehydrogenative C–O coupling of cyclic ethers with 2-hydroxybenzaldehydes using iron carbonyl as catalyst and *tert*-butyl hydroperoxide as oxidant.

The oxidative coupling of 2-hydroxybenzaldehydes **335** with cyclic ethers **336** (1,4-dioxane, tetrahydrofuran) was performed in the presence of the iron carbonyl/*t-*BuOOH system; in these reactions, the ethers served as the solvents (Scheme 76) [274]. The reactions afforded products **337**, leaving the aldehyde group unchanged.

**Scheme 77:**
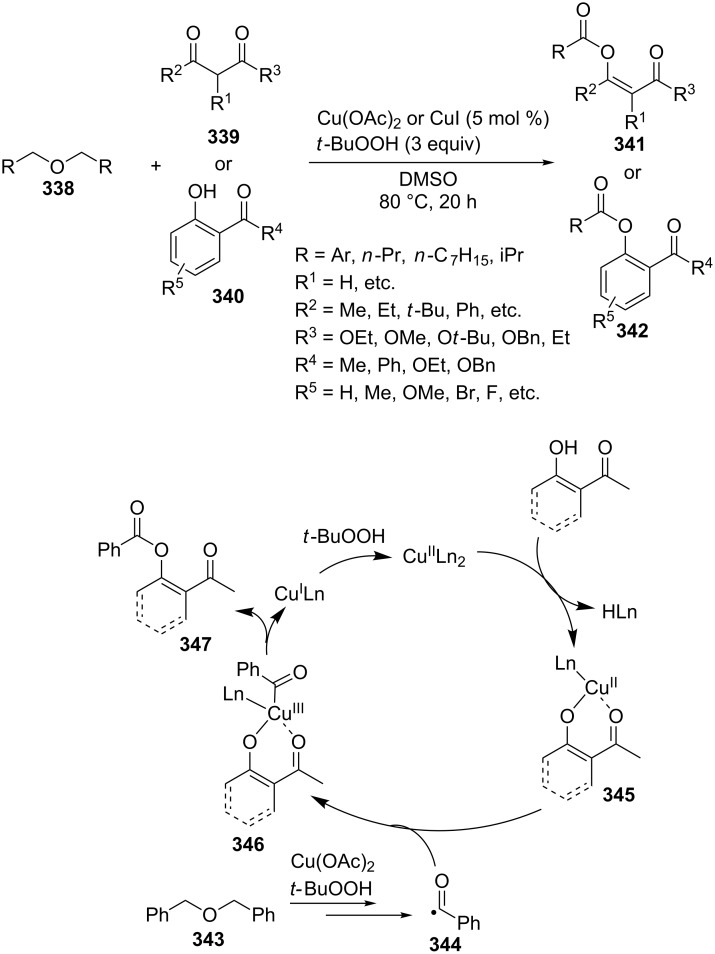
Cross-dehydrogenative C–O coupling of ethers with β-dicarbonyl compounds and phenols using copper catalysts and *tert*-butyl hydroperoxide as oxidant.

Ethers **338** were subjected to oxidative coupling with 1,3-dicarbonyl compounds **339** and *o*-acylphenols **340** using the copper salt/*t-*BuOOH system to prepare esters **341** and **342** (Scheme 77) [275].

In the proposed mechanism (Scheme 77) the reactive benzoyl radical **344** derived from ether **343** by TBHP and a catalytic amount of copper catalyst reacts with complex **345** by single electron transfer to produce Cu(III) complex **346**. Finally, reductive elimination of intermediate **346** affords the desired product **347** and the Cu(I) complex, which can be reoxidized to Cu(II) by TBHP.

**Scheme 78:**
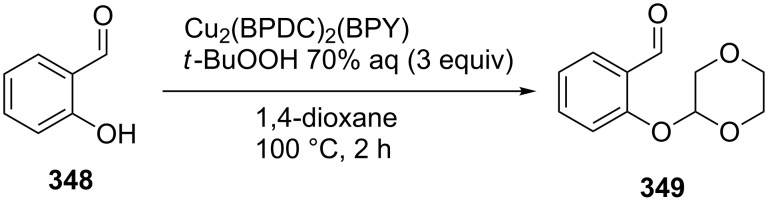
Cross-dehydrogenative C–O coupling of 2-hydroxybenzaldehyde with dioxane catalyzed by Cu_2_(BPDC)_2_(BPY).

Dioxane undergoes C–O oxidative coupling with 2-hydroxybenzaldehyde **348** in the presence of *tert*-butyl hydroperoxide and the metal-organic framework (MOF) Cu_2_(BPDC)_2_(BPY) (BPY = 4,4’-bipyridine, BPDC = 4,4’-biphenyldicarboxylate) as the heterogeneous catalyst [276] (Scheme 78). The yield of product **349** and the reactivity of related CH- and OH-reagents under these reaction conditions were not discussed.

**Scheme 79:**
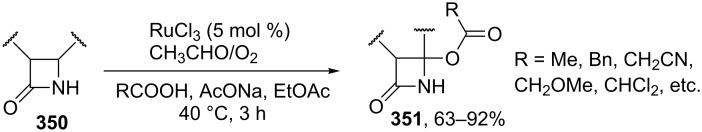
Ruthenium chloride-catalyzed acyloxylation of β-lactams.

The ruthenium chloride-catalyzed acyloxylation of β-lactams **350** with an excess of acids as O-reagents was accomplished using oxygen in the presence of acetaldehyde (Scheme 79). It was hypothesized that the reaction proceeds through the generation of the Ru(V) oxo intermediate, which oxidizes the β-lactam to the carbocation, and the latter is subjected to nucleophilic attack by carboxylic acid to form substituted β-lactam **351** [277].

**Scheme 80:**
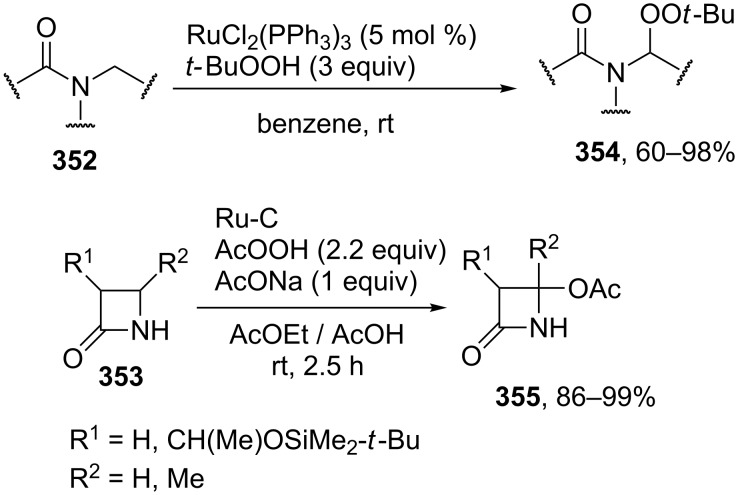
Ruthenium-catalyzed *tert*-butyl peroxydation amides and acetoxylation of β-lactams.

The *tert*-butyl peroxidation (product **354**) or acetoxylation (product **355**) of the methylene groups of amides **352** and **353** adjacent to the nitrogen atom was performed in the presence of a ruthenium-based catalyst using *tert*-butyl hydroperoxide or peracetic acid, respectively [278] (Scheme 80).

**Scheme 81:**
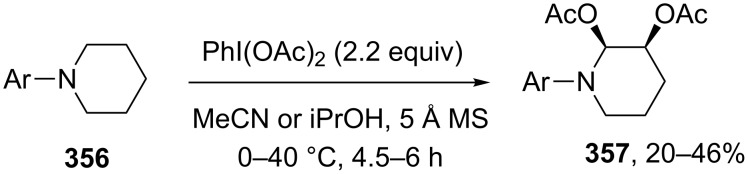
PhI(OAc)_2_-mediated α,β-diacetoxylation of tertiary amines.

The α,β-diacetoxylation of *N*-arylpiperidines **356** with PhI(OAc)_2_ affords products **357** in 20–46% yield. Apparently, the reaction proceeds through the oxidation of amine to enamine [279] (Scheme 81).

**Scheme 82:**
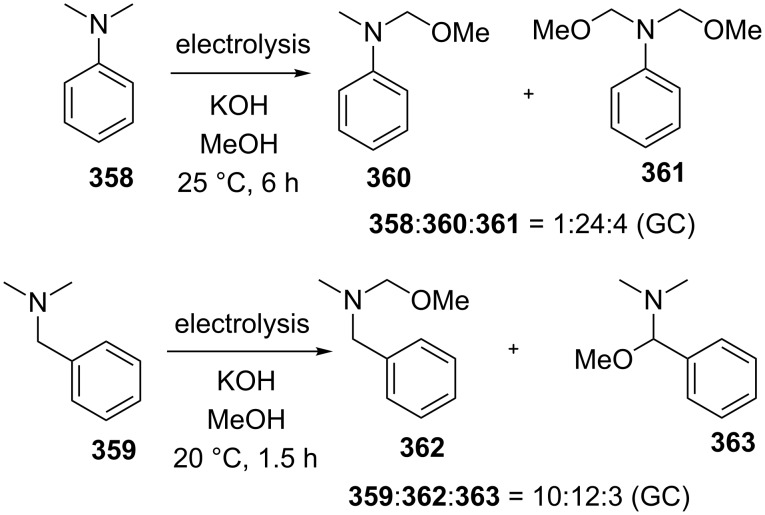
Electrochemical oxidative methoxylation of tertiary amines.

Examples of the alkoxylation of tertiary amines **358** and **359** by their electrooxidation in alcohols were reported in the literature, examples are given in Scheme 82 [280] (the ratio of products **360**–**363** was determined by gas chromatography).

The coupling of *N*-hydroxyphthalimide with tetrahydrofuran (70% yield) and isochromane (71% yield) takes place in the presence of the CuCl/PhI(OAc)_2_ system; CH-reagents are taken in a 10-fold excess (see section 4.4, Scheme 68) [252].

### 6 Other cross-dehydrogenative C–O coupling reactions

**Scheme 83:**
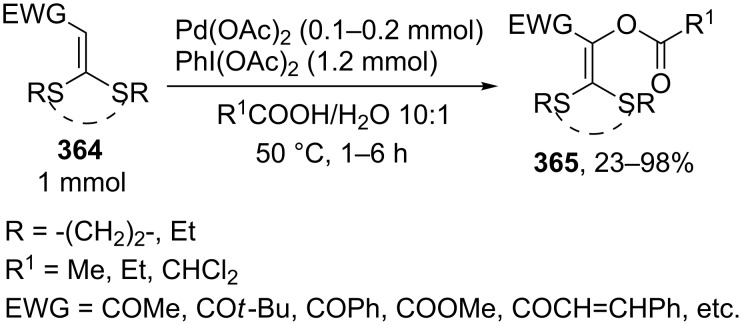
Cross-dehydrogenative C–O coupling of ketene dithioacetals with carboxylic acids in the presence of the Pd(OAc)_2_/PhI(OAc)_2_ system.

Ketene dithioacetals **364** were subjected to acyloxylation in the presence of the Pd(OAc)_2_/PhI(OAc)_2_ system and an excess of carboxylic acids that served as the co-solvents to prepare products **365** (Scheme 83) [281]. The range of carboxylic acids used in this reaction is limited to lower members of the series.

**Scheme 84:**
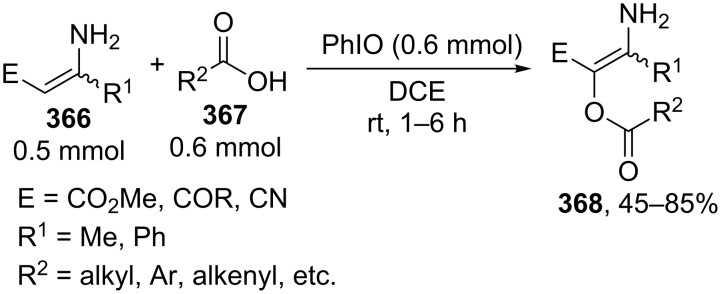
Cross-dehydrogenative C–O coupling of enamides with carboxylic acids using iodosobenzene as oxidant.

The oxidative coupling of enamines containing an electron-withdrawing substituent **366** with carboxylic acids **367** occurs efficiently in the presence of iodosobenzene (Scheme 84); oxidative coupling products **368** were employed in the synthesis of oxazoles [282].

**Scheme 85:**
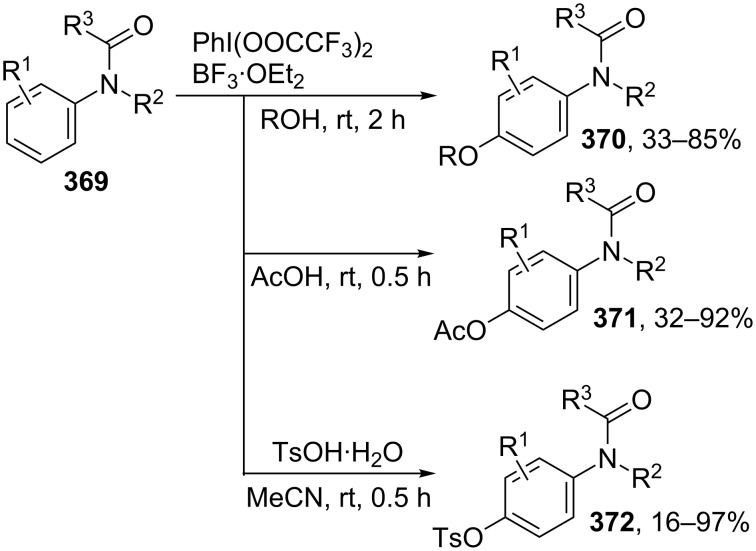
Oxidative alkoxylation, acetoxylation, and tosyloxylation of acylanilides using PhI(O(O)CCF_3_)_2_ in the presence of boron trifluoride.

The oxidative alkoxylation [283], acetoxylation [283], and tosyloxylation [284] of anilides **369** at the *para*-position were accomplished using PhI(OOCCF_3_)_2_ in the presence of boron trifluoride to prepare products **370**–**372**, respectively (Scheme 85); OH-reagents were taken in an excess.

**Scheme 86:**
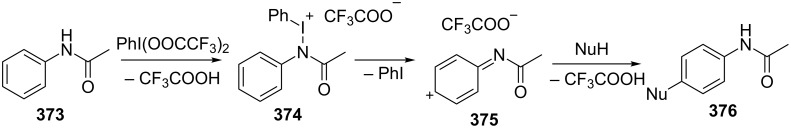
Proposed mechanism of the oxidative C–O coupling of actetanilide with O-nucleophiles in the presence of (di(trifluoroacetoxy)iodo)benzene.

The following mechanism was proposed. Anilide **373** is subjected to electrophilic attack by (di(trifluoroacetoxy)iodo)benzene to form intermediate **374** followed by the elimination of phenyl iodide from **374** to give cation **375**, which is subjected to nucleophilic attack (ROH, AcOH, or TsOH) affording coupling product **376** (Scheme 86).

**Scheme 87:**
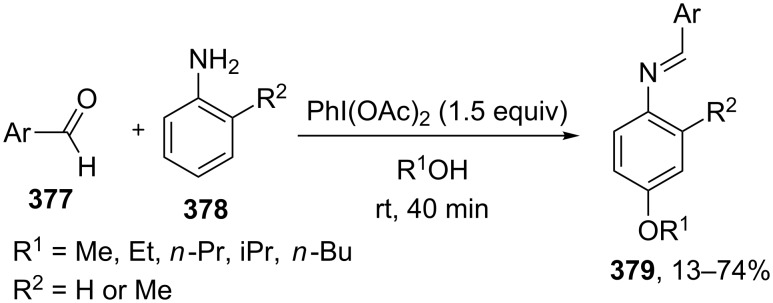
Three-component coupling of aldehydes, anilines and alcohols involving oxidative intermolecular C–O bond formation.

In the presence of PhI(OAc)_2_, aldehydes **377**, anilines **378** and alcohols undergo three-component coupling to produce alkoxyl-substituted *N*-arylimines **379** (Scheme 87). The preliminary mechanism investigations revealed that the transformation proceeds via imines as intermediates. Reactions were performed in the medium of alcohol (OH-reagent) at room temperature [285].

**Scheme 88:**
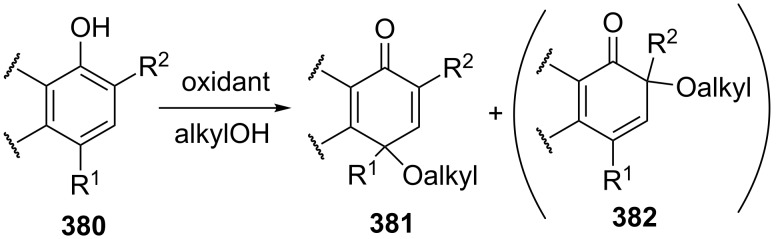
Oxidative coupling of phenols with alcohols.

Various oxidants, such as (diacetoxyiodo)benzene [286–288], PbCl_2_/HClO_4_, NaIO_3_/HClO_4_, Br_2_/*N*-iodosuccinimide, or I_2_/30% aq H_2_O_2_ [289], were employed in the oxidative coupling of phenols **380** with alcohols serving as the solvents (Scheme 88) to prepare non-aromatic products **381** or **382**.

**Scheme 89:**
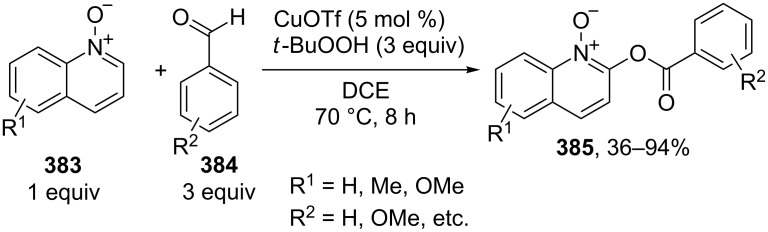
2-Acyloxylation of quinoline *N*-oxides with arylaldehydes in the presence of the CuOTf/*t*-BuOOH system.

The 2-acyloxylation of quinoline *N*-oxides **383** with arylaldehydes **384** in the presence of the CuOTf/*t*-BuOOH system afforded products **385** (Scheme 89) [290]. Benzaldehyde and aromatic aldehydes containing electron-donating groups were employed in the coupling. In addition to aromatic aldehydes, the coupling was performed with cyclohexanecarbaldehyde. It is suggested that the reaction proceeds through a radical mechanism.

**Scheme 90:**
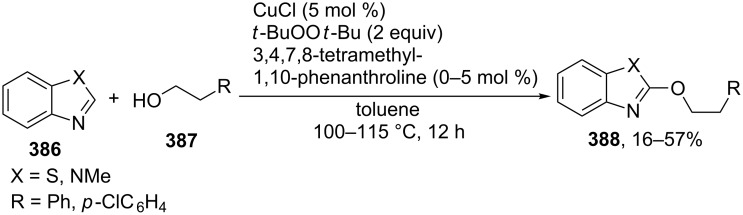
Cross-dehydrogenative C–O coupling of azoles with primary alcohols.

The oxidative coupling of primary alcohols **387** with azoles **386** was performed in the presence of the CuCl/(*t*-BuO)_2_ system (Scheme 90) to prepare products **388** in 16–57% yield [291]. It was hypothesized that the C–O bond is formed as a result of the reductive elimination of coupling product **388** from the copper(III) complex.

**Scheme 91:**
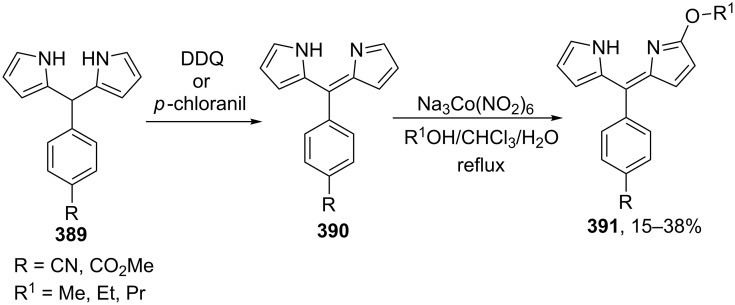
Oxidation of dipyrroles to dipyrrins and subsequent oxidative alkoxylation in the presence of Na_3_Co(NO_2_)_6_.

Alkoxylated heterocycles **391** were obtained in an excess of alcohol in the presence of Na_3_Co(NO_2_)_6_ from dipyrrins **390**, which were generated from dipyrroles **389** (Scheme 91) [292].

1,2- and 1,4-naphthoquinones undergo alkoxylation in an alcoholic medium in the presence of transition metal salts [293], I_2_/CeCl_3_ [294], and HgO [295].

Alkanes are rarely used in the cross-dehydrogenative C–O coupling because of low reactivity of C–H bonds. An example is the trifluoroacetoxylation of cyclohexane, cycloheptane, cyclooctane, and adamantane in trifluoroacetic acid in the presence of peracetic acid [296] or hydrogen peroxide [297–299] with the addition of transition metal salts (Rh, Ru, Pd, Pt, Fe) [296–297] or in the absence of metal compounds [298–299]; the reactions were usually accomplished at room temperature for a few hours.

**Scheme 92:**
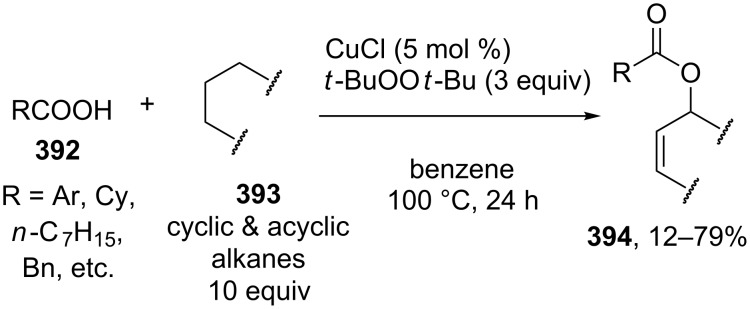
Oxidative dehydrogenative carboxylation of alkanes and cycloalkanes to allylic esters.

Copper-catalyzed oxidative dehydrogenative carboxylation of unactivated alkanes **393** with various aromatic acids **392** to produce the corresponding allylic esters **394** was reported recently [300] (Scheme 92). Mechanistic studies allowed proposing a mechanism involving the generation of an allyl radical via the formation of alkene from the starting alkane. Related oxidative C–O coupling of aromatic aldehydes [169] and methylarenes [229] with cycloalkanes using a Cu(OAc)_2_/*t*-BuOOH system were considered in sections 2.5 (Scheme 38) and 4.1 (Scheme 56), respectively.

**Scheme 93:**
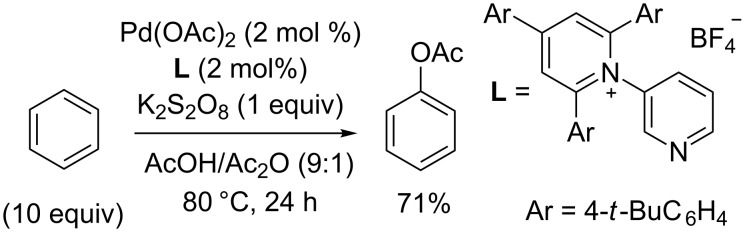
Pd-catalyzed acetoxylation of benzene.

Acetoxylation of benzene was achieved employing Pd(OAc)_2_/K_2_S_2_O_8_ oxidative system in AcOH/Ac_2_O mixture in the presence of a pyridinium-substituted pyridine ligand [301] (Scheme 93). It was proposed that a key role for this cationic ligand is to serve as a phase transfer catalyst to bring poorly soluble S_2_O_8_^2−^ into contact with the Pd catalyst.

The electrochemical cross-dehydrogenative C–O coupling, in which the O-reagent serves as the solvent, was described in the literature. For example, the methoxylation of methylarenes, amides, phenols, and other compounds was performed in methanol using bases immobilized on a solid support [302].

## Conclusion

The analysis of the published data shows that oxidative C–O coupling reactions attract an increasing interest of organic chemists in recent years. In the past two decades, a considerable body of experimental data has been accumulated. Compared with other types of coupling, the oxidative C–O coupling is less well-studied despite the fact that the C–O–R group is rather commonly present in various classes of organic compounds, and a large number of diverse O-reagents are available for the coupling.

The drawback of the majority of the available methods for the cross-dehydrogenative C–O coupling, which limits their application to the coupling of two expensive complex compounds, is the use of an excess of one of the starting components, the C- or O-reagent. Besides, the synthesis often requires high temperature and long time to be completed.

The main goals in the development of the cross-dehydrogenative C–O coupling are as follows: (1) a search for new reactions involving various oxidative systems, C- and O-reagents; (2) the development of methods, which do not require an excess of the C- or O-reagent; (3) the development of methods based on available, convenient, and safe oxidants; (4) a decrease in the reaction temperature and time; (5) investigations of the mechanisms of the oxidative coupling with the aim of predicting conditions necessary for the efficient synthesis.
